# Untangling the Evolution of American Wild Grapes: Admixed Species and How to Find Them

**DOI:** 10.3389/fpls.2019.01814

**Published:** 2020-02-07

**Authors:** Giovanni Zecca, Massimo Labra, Fabrizio Grassi

**Affiliations:** ^1^ Department of Biotechnology and Biosciences, University of Milano-Bicocca, Milano, Italy; ^2^ Department of Biology, University of Bari, Bari, Italy

**Keywords:** climate changes, gene flow, hybrids, introgression, migrations, phylogeny, TreeMix, *Vitis*

## Abstract

Natural hybridization and introgression are central evolutionary processes in grape genus (*Vitis*). On the other hand, the interspecific relationships among grapes, the directionality of the inferred admixture events and the parents of hybrids are not yet completely clarified. The grapes are economically important crops characterized by tendrils used to climb on the trees and the fruits harvested by humans especially for the consumption or to produce wines and liquors. The American grapes (ca. 30 species) are recognized as an important resource because they show biotic and abiotic resistances. We analyzed 3,885 genome-wide SNPs from 31 American *Vitis* species using the TreeMix software combined with the *f*3 and *f*4 tests. This approach allowed us to infer phylogenetic relationships and to explore the natural admixture among taxa. Our results confirmed the existence of all hybrid species recognized in literature (*V. x champinii, V. x doaniana, V. x novae-angliae, and V. x slavinii*), identifying their most likely parent species and provided evidence of additional gene flows between distantly related species. We discuss our results to elucidate the origin of American wild grapes, demonstrating that admixture events have ancient origins. We observe that gene flows have involved taxa currently spread through the southern regions of North America. Consequently, we propose that glacial cycles could have triggered the contact between interfertile taxa promoting local hybridization events. We conclude by discussing the phylogenetic implications of our findings and showing that TreeMix can provide novel insights into the evolutionary history of grapes.

## Introduction

Hybridization is a natural process of evolution observed in plants and animals. To find pure hybrids in nature is considered occasional, whereas introgression events are more frequent (Mallet, 2005; Folk et al., 2018). Gene introgression is a long-term process, mediated by the transfer of genes between taxa through repeated backcrosses (Anderson and Hubricht, 1938; Harrison and Larson, 2014). It can have deleterious consequences on the genetic structure and conservation of wild species, analogous to the ecological consequences caused by the introduction of nonnative taxa (Allendorf et al., 2001; Mallet, 2005). Continued and unidirectional introgression may lead to a crescent depauperating of genetic variability especially if the gene flow involves species spread over restricted areas (Grassi et al., 2006b; Beatty et al., 2015; Lau and Jacobs, 2017). On the other hand, hybridization and introgression can also be a source of genetic variation accelerating adaptive radiation and speciation when the new alleles are conserved by natural selection (Mallet, 2007; Ma et al., 2018b). Adaptive introgression of favourable genes has been observed in plants showing new biotic or abiotic resistances (Suarez-Gonzalez et al., 2018). It is thought indeed that nearly a quarter of the species of flowering plants have experienced natural hybridization and introgression with other species (Mallet, 2007; Ma et al., 2018b).

The grapes (i.e., genus *Vitis* L.) are a complex of species with wide morphological variability adapted to a range of climatic conditions (Callen et al., 2016; Chitwood et al., 2016). The genus consists of ca. 70 species mainly distributed in the northern hemisphere (Zecca et al., 2012; Liu et al., 2016; Ma et al., 2018a) and is characterized by tendrils used to climb for tens of meters on the crown of adjacent trees. Morphological and molecular data confirm a subdivision in two subgenera, named *Muscadinia* (Planch.; 2n = 40) and *Vitis* (2n = 38). North America, represented by over 30 species, has been proposed as the centre of origin for the genus (Zecca et al., 2012; Liu et al., 2016; Moore and Wen, 2016). Today most of American taxa are distributed in the Eastern and Southern States, with geographical ranges that partially overlap (Heinitz et al., 2019). These species have attracted increasing interest because several native North American grapes harbour resistance or tolerance genes toward both diseases and environmental stresses (Töpfer et al., 2011; Heinitz et al., 2019). Another remarkable feature of American grapes is that hybrid origins have been suggested for several of them. Comeaux et al. (1987), acknowledged that natural hybrids were occasionally observed and described in the past, but proposed that geographical and phenological barriers had sufficiently prevented hybridization of American wild grapes (Munson, 1909; Bailey, 1934; Olmo and Koyama, 1980). Conversely, Moore (1991) and Mullins et al. (1992) observed that hybridization was more common than thought, mostly in Texas where several taxa occur (Pittcock, 1997). In particular, the species belonging to the series *Ripariae* (*V. acerifolia* Raf.*, V. riparia* Michx., and *V. rupestris* Scheele) as described in Moore (1991) were supposed to be involved in several hybridization processes. Miller et al. (2013) suggested that *V. acerifolia* Raf. itself might be of hybrid origin. Today at least three named hybrid taxa native of North America have been proposed, *V.* x *champinii* Planch., *V.* x *doaniana* Munson ex Viala, and *V. x novae-angliae* Fernald, and a forth taxa, *V.* x *slavinii* Reheder, has been suggested as another likely natural hybrid species (Moore and Wen, 2016; USDA, 2019).

Phylogenetic reconstructions of the *Vitis* genus, based on nuclear and plastid DNA sequences, have shown discrepancies in genetic relationships (Tröndle et al., 2010; Péros et al., 2011; Zecca et al., 2012; Miller et al., 2013; Wan et al., 2013; Liu et al., 2016; Klein et al., 2018; Wen et al., 2018). Thought other factors such as recent times of radiation, low substitution rates and incomplete lineage sorting may have obscured the real phylogenetic signal (Zecca et al., 2012; Wan et al., 2013; Wen et al., 2018; Zecca et al., 2019), natural hybridization and introgression have been proposed as central evolutionary processes to explain the observed conflicting results (Péros et al., 2011; Zecca et al., 2012; Aradhya et al., 2013; Miller et al., 2013; Wan et al., 2013; Moore and Wen, 2016). Molecular data have been applied to confirm hybridization of Asian and European grapes (Zecca et al., 2010; Goto-Yamamoto et al., 2015; Ma et al., 2018b) and to show extensive reticulate evolution among grapes (Wan et al., 2013). In parallel however, disagreement between authors about the directionality of the inferred admixture events as well as about the identification of natural hybrids and of their ancestry has increased, confirming that the interspecific relationships among grapes have not yet been completely clarified (Pinto-Maglio et al., 2010; Miller et al., 2013; Dangl et al., 2015; Goto-Yamamoto et al., 2015; Klein et al., 2018; Ma et al., 2018b; Heinitz et al., 2019; Liang et al., 2019; Zecca et al., 2019).

Moreover, when also cultivated grapes are taken into consideration, the overall picture appears even more complex because of the lack of significant genetic barriers between wild and cultivated grapes. While several species (e.g., *V. amurensis* Rupr., *V. labrusca* L., *V. aestivalis* Michx., *V. mustangensis* Buckley, *V. riparia* Michx., *V. rotundifolia* Michx.) are harvested for the consumption and for the production of grape juices, jam, jelly, raisins, liquors, and wine, the domesticated grapevine (i.e., *V. vinifera* L.) is certainly the most widespread and economically important species of the genus (De Mattia et al., 2008; Töpfer et al., 2011). Recent genome analyses however, have shown the high vulnerability of the domesticated grapevine, suggesting that prolonged vegetative propagation combined with limited sexual reproduction, have dramatically reduced its resistance to pathogens and adverse environmental conditions (Myles et al., 2011). The problem has been well known for a long time. An intensive breeding program had been planned from the middle of the 19th century due to the spread of phylloxera (*Daktulosphaira vitifoliae* Fitch.) and other pests in Europe (Millardet, 1880; Töpfer et al., 2011). About 800,000 ha of vineyards were destroyed in France in only 15 years, with serious repercussions also on the demography of wild populations (Töpfer et al., 2011; Zecca and Grassi, 2013). Since then, several native North American grapes have been employed by plant breeders to produce new rootstocks resistant to pathogens and many *V. vinifera* varieties have been exploited to produce new cultivars characterized by the good quality of fruit and resistance to biotic and abiotic stress (This et al., 2006; Töpfer et al., 2011). Still today, new rootstocks are being developed to address climate changes, using wild species characterized by drought and salt tolerances (Heinitz et al., 2019). In this context, many interspecific hybrids have also been developed, especially crossing wild American grapes with *V. vinifera*. American Hybrids is the term traditionally used to indicate numerous North American commercial cultivars that show *V. vinifera* ancestry. Molecular studies carried out on the most widespread American Hybrids have shown recent and frequent backcrosses with wild grapes (Sawler et al., 2013; Migicovsky et al., 2016). Since wild populations are still not sufficiently studied in their ecosystems (Pavek et al., 2003; Everhart, 2010), this eventuality may involve the actual risk of their genetic resources dwindling. For example, species like *V. monticola* Buckley and *V. cinera* var. *helleri* (L. H. Bailey) M. O. Moore are confined to narrow natural ranges today and are seriously threatened by human activities, such as grazing and the increasing use of herbicide along roadways, and by the diffusion of invasive taxa (Comeaux et al., 1987; Pavek et al., 2001; Heinitz et al., 2019). Rootstocks, cultivated hybrids or cultivars which have escaped from cultivation and naturalized in the wild environment, especially due to vegetative reproduction and the spread of seeds by birds (Arrigo and Arnold, 2007; Zecca et al., 2010; Ardenghi et al., 2015) can cause an uncontrolled transfer of nonnative genes to wild populations, with severe consequences for the genetic variability and biodiversity of ecosystems (Arnold et al., 2005; Grassi et al., 2006a; Dangl et al., 2015).

Nowadays, accessibility to genomic data has greatly improved our ability to investigate relationships among species, but results of hybridization and introgression still remain hard to untangle. Interspecific gene flow challenges the strictly tree-like paradigm of evolution assumed by classical phylogenetic models (Leaché et al., 2014; Folk et al., 2018). Admixture events may have occurred recently or far in the past, for example due to the continuing climate changes (Zecca et al., 2012; Wan et al., 2013; Klein et al., 2018) and can thereby affect all parts of a tree, not just recently diverged tips (Mallet et al., 2016). Traces of ancient admixture are more difficult to reveal than recent hybridizations, consequently, the real level of introgression may be underestimated (Folk et al., 2018). On the other hand, the wide backcross selection operated by plant breeders have produced several hundreds of cultivars that, if analyzed in a phylogenetic context, can produce an overestimation of the level of introgression, deranging the real genetic relationships (Klein et al., 2018). Thus, modern tools require the integration of population genetic and phylogenetic approaches to coestimate speciation tree and interspecific gene flows using genomic data. At the same time, when the goal is to study the evolution of wild species, it is necessary to undertake an accurate sample inspection before running the analyses to minimize the influence of human driven effects.

In this study, we analyzed 459 accessions from 31 *Vitis* species and 3,885 genome-wide SNPs using the model implemented in TreeMix to test natural admixture among native wild grapes of America. TreeMix has been used to infer recent and ancient introgressions in wild and domesticated species (Brandvain et al., 2014; Flowers et al., 2019). Differently from other methods, TreeMix was developed to address directly historical relationships (Pickrell and Pritchard, 2012). This method has the advantage of being applicable to several taxa simultaneously using a graph representation that allows to show both splits and migration events. Treemix model is a simplification of the migration process and, as suggested by authors, it works best when gene flow is restricted to a relatively short time period and when the history of the species is largely tree-like (Pickrell and Pritchard, 2012).

In particular we aimed to: (*i*) substantiate the hybrid origin of *V.* x *champinii*, *V.* x *doaniana*, *V.* x *novae-angliae,* and *V.* x *slavinii* and clarify their ancestry; (*ii*) assess whether additional admixture events have contributed to shape the current diversity observed in American wild grapes; (*iii*) clarify the phylogenetic relationships between taxa. In doing so, we applied a multi-step procedure to minimize the impact of gene flow driven by human activities and we confirmed TreeMix results using the less parametrized *f*3 and *f*4 tests of treeness. We also provide all scripts used in our TreeMix analysis pipeline. Finally, we discuss the contribution of our results to elucidate the evolutionary history of the American wild grapes and the implications of these findings for the conservation of grapes.

## Materials and Methods

### Samples Selection

Row data, including 6,114 SNPs in 1,817 *Vitis* samples, were generated by Myles et al. (2010; 2011) through the custom Illumina Vitis9KSNP array and were downloaded from the FigShare repository (https://doi.org/10.6084/m9.figshare.9784325.v1). While row data includes species from both Old and New Worlds, for the purpose of this work we focused our attention on wild American species only. In addition, to avoid potentially confounding signals, we endeavoured to minimize the impact of gene flow driven by human activities. To achieve our goal, we applied a multi-step strategy to samples selection. First, a*ll* accessions named “*hybrid,*” “*sp.,*” *Vitis* ×*andersonii*, *Vitis ×bourquiniana* or *V. vinifera* subsp. *sylvestris* were discarded from the original dataset as well as all Asian species. However, 36 accessions of *V. vinifera* subsp. *vinifera* were retained in the subsequent analysis (**Supplementary Table S1**) to exclude a possible gene flow between cultivars and wild American species. Second, 100 independent Maximum Likelihood (ML) searches were performed in RAxML-HPC2 v. 8.2.10 (Stamatakis, 2014) under the GTRCAT model of substitution, with the number of distinct rate categories (-*c*) set to 25, the best rearrangement setting (-*i*) determined automatically by RAxML and the *–asc-corr* option set to *lewis*. A random maximum parsimony starting tree was used to initialize each ML tree search. The best-scoring ML-tree was carefully inspected and American species with anomalous placement due to a possible hybridization with *V. vinifera* or to possible cases of mislabeling or misidentification as well as accessions with unknown accession number were discarded. Third, based on information available in literature we excluded from the downstream analysis all accessions identified as: American Hybrids, cultivar of interspecific ancestries, hybrids of *V. vinifera* ancestry, tetraploids or specimens donated from Eurasian countries. All remaining accessions were named according to the nomenclature proposed by GRIN Taxonomy (https://npgsweb.ars-grin.gov; accessed February 9, 2017).

### Data Preparation and Linkage Disequilibrium Assessment

Data cleaning was conducted using PLINK v. 1.07 (Purcell et al., 2007). First, we discarded all SNPs not assigned and anchored to locations on chromosomes 1 to 19. Then, accessions and SNPs with >20% missing data were removed, followed by SNPs with minor allele frequency (MAF) < 0.01. Because TreeMix software (see next paragraph) is sensitive to linkage disequilibrium (LD) and because previous evaluations of LD decay in grapes yielded inconsistent results (Liang et al., 2019 and references therein), before running the analysis, we explored the LD decay within species using PLINK. Data were split by species and each subset was further filtered using the following options: *–mind* 0.2 and *–maf* 0.01. For species with at least 10 accessions and 600 intraspecific SNPs retained after data cleaning, pairwise LD was calculated for all SNP pairs that were no more than 500 kb apart (*–ld-window-kb* 500), using r^2^ values as proxy. LD values were sorted by their inter-SNP distance, and then the median r^2^ was calculated in sequential bins of 75 pairwise SNP comparisons and plotted against the mean physical distance of each bin. By inspecting LD values, we identified two accessions of *V. labrusca* that produced anomalous signals with respect to their conspecifics. We left out these two samples from the following analysis because both were clustered together with other excluded accessions in the ML-tree. Based on the visual inspection of LD decay graphs, we derived an inter-SNPs distance threshold that far exceeds the extent of the observed LD, as recommended in the software manual. TreeMix, however, does not allow a physical distance to be specified to account for linkage disequilibrium, but instead it allows the user to group together contiguous SNPs in blocks of equal size. To identify the appropriate block size, we developed the following procedure. We started with five SNPs per block and we computed the physical position of the midpoint of each block. When a block was straddling two chromosomes, it was split into two parts, and we calculated two midpoints using SNPs from the first or the second chromosome only. Then, for each chromosome, we computed the distance between the midpoints of consecutive blocks and we compared these values with the previously identified distance threshold. We iterated this procedure adding 5 SNPs per block each time until all calculated distances were found to be larger than the established threshold. Since we applied a very conservative distance threshold, we considered the block size determined in this way as adequate to account for most of the linkage disequilibrium present among loci. The custom R scripts MdMn_LD, mMd_LD, and BlockDistByChr used to carry out these analyses are described in **Supplementary file S1** (R Core Team, 2013).

### Treemix Analysis

The TreeMix v. 1.12 software (Pickrell and Pritchard, 2012) was used to reconstruct the ML tree of American wild grapes and to model gene flow between species. Stratified allele frequencies output from PLINK were converted into TreeMix format using the plink2treemix.py script included in the software release. We performed 400 independent ML searches in TreeMix with the following settings: SNP block size set to 20 (option -*k*), global tree rearrangements option activated (option -*global*), trees rooted using *V. rotundifolia* as outgroup (option -*root*), and the *-noss* option activated to avoid sample size overcorrection. The choice of *V. rotundifolia* as outgroup is consistent with previous studies that identified muscadines as the sister species to subgenus *Vitis* (Tröndle et al, 2010; Péros et al., 2011; Zecca et al., 2012; Wan et al., 2013; Moore and Wen, 2016). We used the custom R script cfTrees (**Supplementary file S1**) to filter results automatically based on their likelihood values, remove duplicates from the list of best-scoring ML trees, compute the Robinson and Foulds (R–F) distance (Robinson and Foulds, 1981) among the remaining trees and generate a strict consensus tree from them. Since only two best-scoring ML trees were retained after removing duplicates, we used these trees to compute the percentage of variance explained by the model under the assumption of a completely tree-like evolutionary history. Both best-scoring ML trees were used as a starting point to model gene flow between species in the following analyses. Initially, we explored the effect of adding migration events sequentially by testing migration edges from 4 to 9 with five different random seeds each time. Although the percentage of variance explained by the model increased with the number of migration events, their estimated p-values became nonsignificant after the eighth migration. Accordingly, 40 final runs, starting from as many random seeds, were performed with the following settings: -*k* 20, -*m* 9, -*global*, -*root V. rotundifolia,* -*noss.* The two estimated ML trees were used as starting point for fitting migration events. Five hundred bootstrap replicates were performed resampling over blocks of contiguous SNPs and bootstrap support, values were mapped on nodes using the *phytools* R package (Revell, 2012). We used the method described in **Supplementary file S1** to summarize the inferred migrations and to calculate an index of support for each migration edge identified by TreeMix. Although bootstrap-based, this index is not the same as the bootstrap supports shown on phylogenetic trees. Therefore, to avoid confusion, hereafter we refer to the former as migration support (MS).

### *f*3 and *f*4 Statistics

To confirm TreeMix results we used the less parametrized three- and four-population tests (*f*3 and *f*4) of treeness (Reich et al., 2009; Patterson et al., 2012) as implemented in the programs *threepop* and *fourpop* included in the TreeMix package. For the three-population test, the null hypothesis is that the *f*3 is nonnegative (which corresponds to a tree-like evolutionary history); whereas negative values indicate that, the null hypothesis is rejected in favour of the alternative hypothesis (i.e., a more complex admixture graph). Given three taxa *X*, *Y* and *W*, a significantly negative value for the test *f*3(*X*; *Y*, *W*) implies that taxon *X* is admixed. For the four-population test, the expectation of *f*4 is zero under the null hypothesis whereas departures from zero indicate the presence of admixture. Thus, given four taxa *X*, *Y*, *W,* and *Z*, a significantly nonzero value for the test *f*4(*X*, *Y*; *W*, *Z*) indicates gene flow in the tree. While originally addressed to populations, this statistic has shown to be effective also with species that diverged several million years ago to distinguish between introgression and incomplete lineage sorting. In the absence of introgression the *f*4-statistic is expected to be zero even in the presence of incomplete lineage sorting, allowing us to deduce introgression between species when its value is significantly different from zero (Meyer et al., 2017). Z-score were reported for these tests and standard errors of *f*3 and *f*4 statistics were computed using a block jackknifing procedure with data split into blocks of 20 SNPs (Pickrell and Pritchard, 2012). The three-population statistic can also be interpreted in terms of shared branch length, the so-called ‘outgroup’ statistic *f*3 (Raghavan et al., 2014). In this context, if *T* is a taxon of interest, the idea is to find the most closely related taxon from a reference set of *k* extant taxa {*R_i_*, *i* = 1, 2,… k}, given an outgroup taxon *O* diverging from both the taxon of interest and the reference set. Thus, the ‘outgroup’ *f*3 statistic actually measures the shared drift between *T* and the taxa in the reference set, given the outgroup *O*. This statistic is always positive and high values imply close relatedness between *T* and *R.* However, values have no absolute meaning and can be interpreted only in the context of the reference set *R_i_.* Therefore, Z-score were reported for these tests as described above and the |Z_diff_| score was taken as the strength of evidence in favour of one taxon over the other (Flegontov et al., 2016). We applied the Holm-Bonferroni method (Holm, 1979) to control the familywise error rate for multiple *f*3 and *f*4 tests.

## Results

### TreeMix Analysis

**Figure 1:**
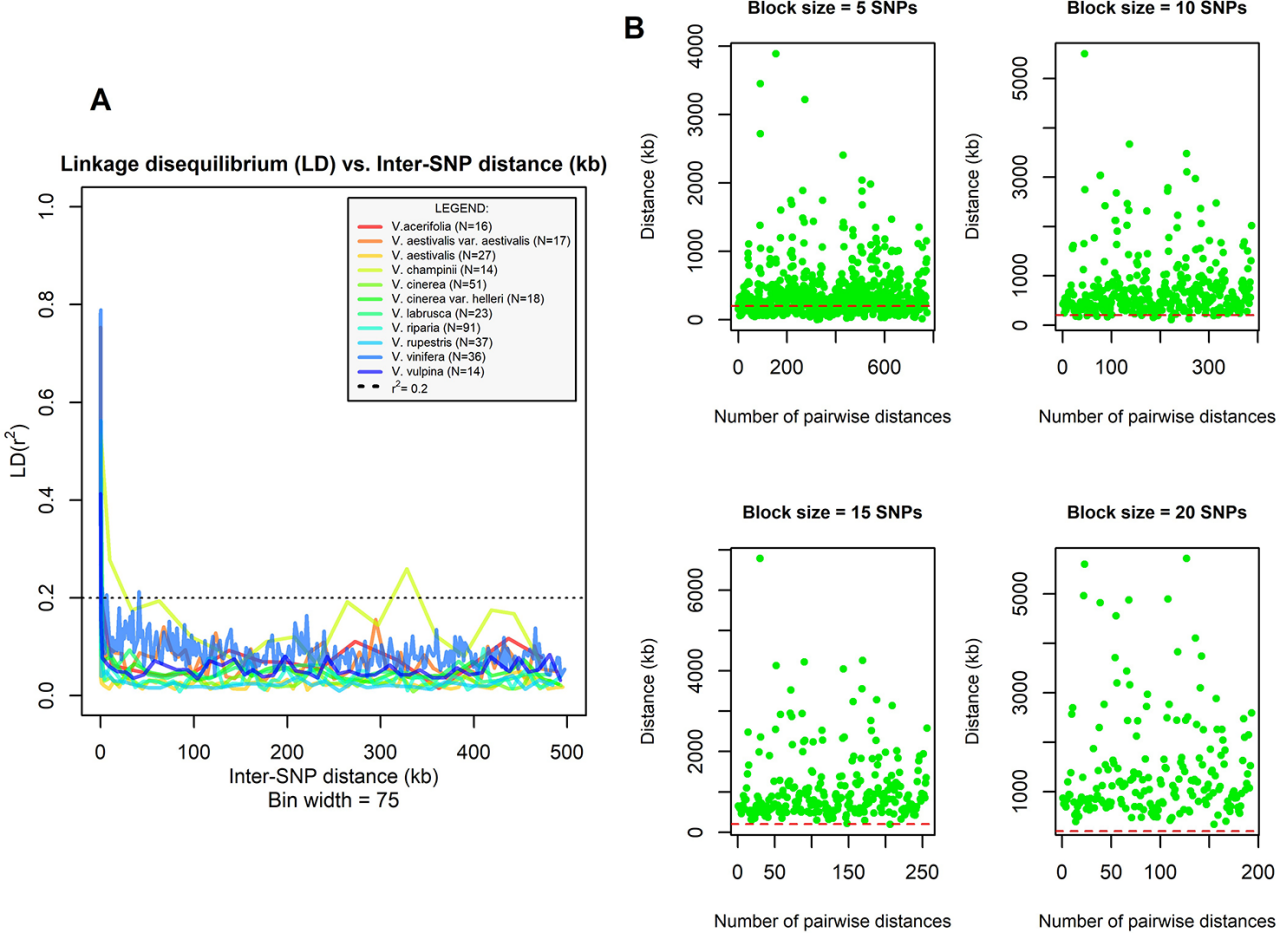
**(A)** Linkage disequilibrium (LD) decay in ten grape species. The median r^2^ calculated in bins of 75 pairwise SNP comparisons was plotted against the mean physical distance of each bin (kb). Sample size for each species is shown in figure. Dotted line indicates r^2^ = 0.2. **(B)** Multistep procedure followed to identify the appropriate SNP block size used in TreeMix analysis. The SNP block size tested at each step is shown above plots. The physical distance (kb) between midpoints of consecutive blocks placed on the same chromosome is shown on y-axis while the x-axis shows the number of pairwise distances computed at each step. The dashed red line represents the assumed distance threshold (i.e., 200 kb).

**Figure 2:**
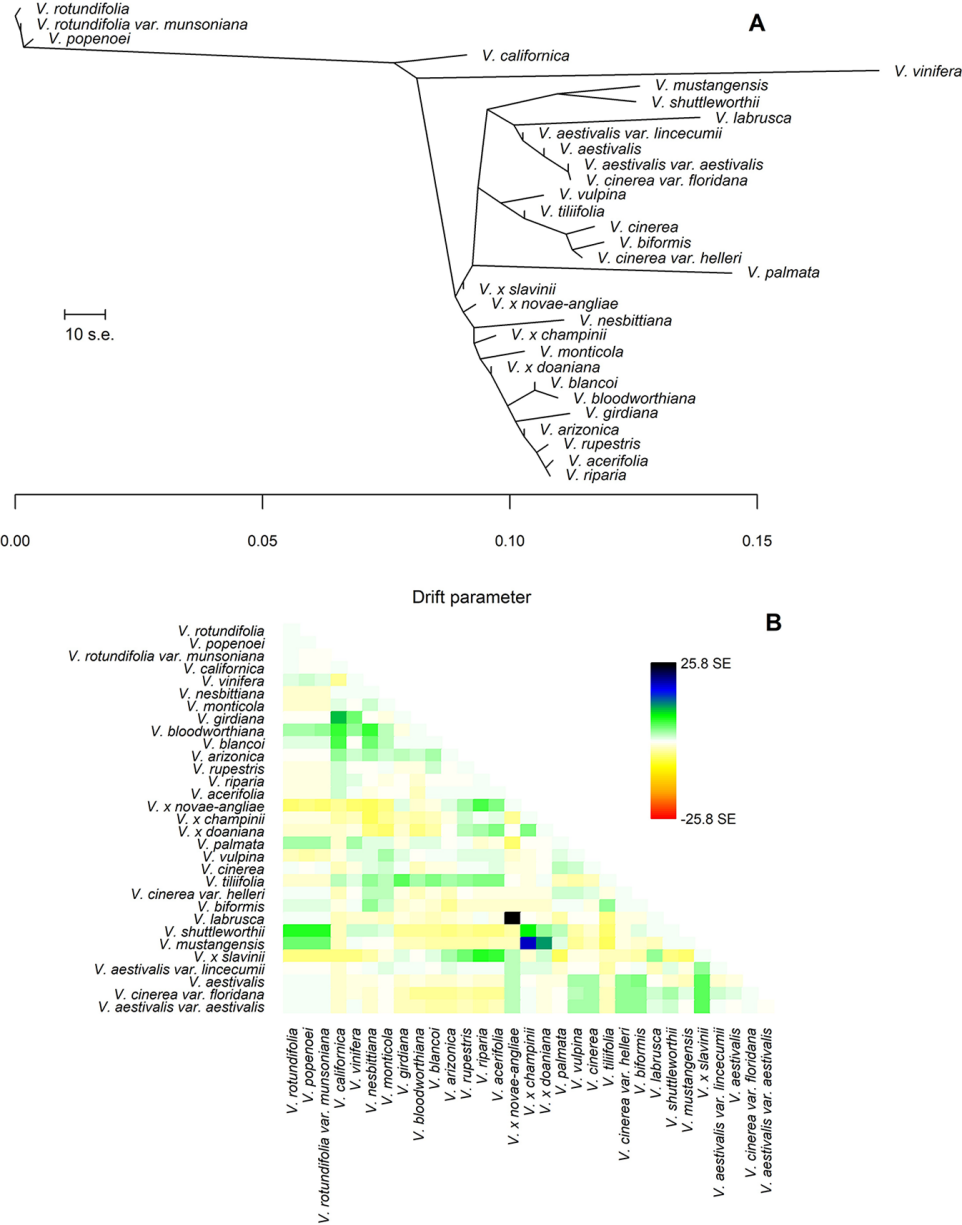
**(A)** The Maximum Likelihood (ML)-tree inferred by TreeMix under the strictly bifurcating model. The scale bar shows ten times the average standard error (s.e.) of the entries in the sample covariance matrix. Drift parameter is shown on the x-axis. **(B)** Scaled residuals from the fit of the model to the data. Without migration events 91.7% of the variance in relatedness between taxa was explained by the tree. Colors are described in the palette on the right.

**Figure 3:**
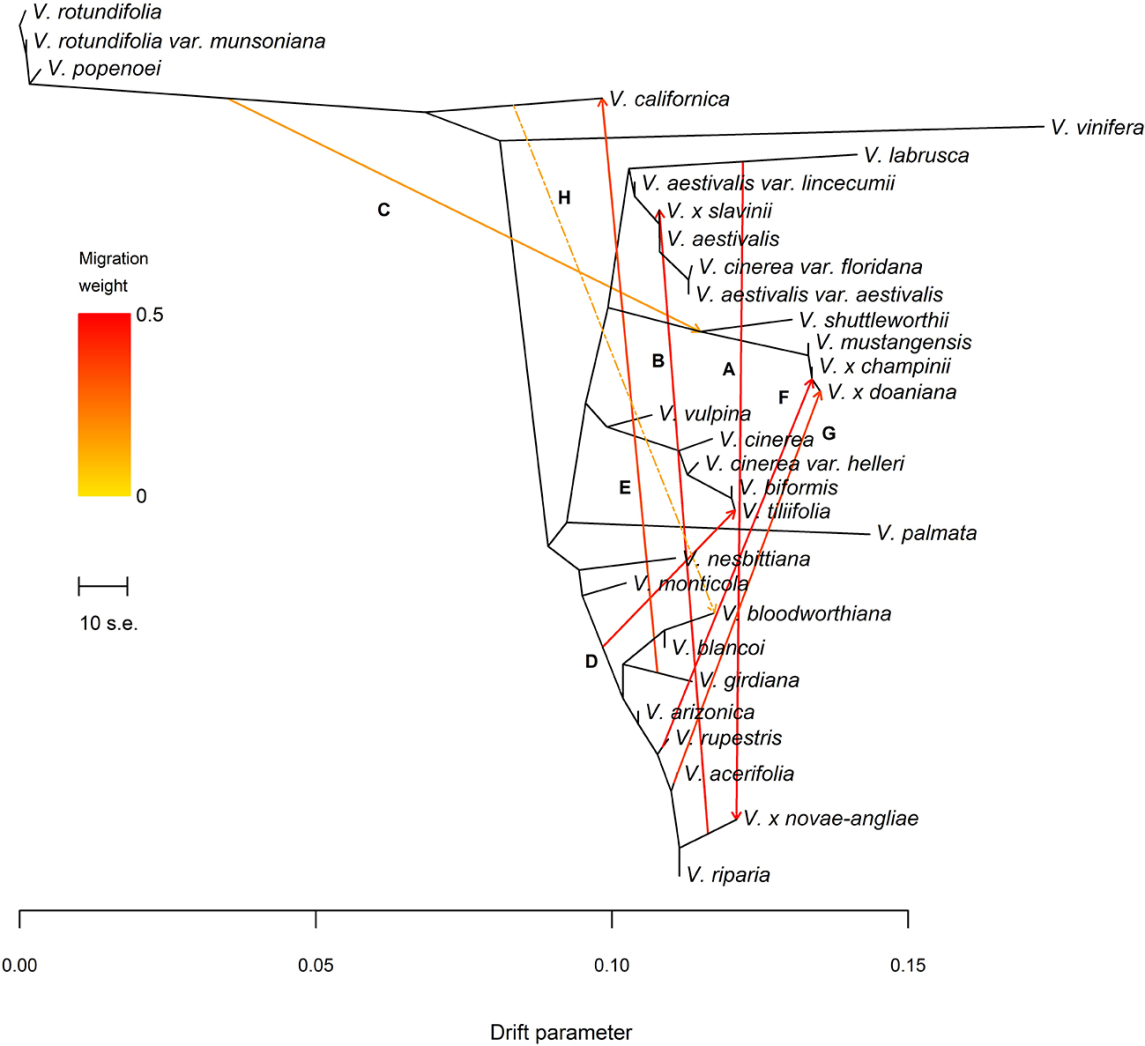
**(A)** Maximum Likelihood (ML)-tree inferred by TreeMix allowing eight migration events. Letters A–H refer to migration edges as defined in **Table 1**. Migration H, not confirmed by the *f*4 statistic after the Holm-Bonferroni correction (see **Table 3**), is shown using a two-dashed line. Migration arrows are colored according to their weight and colors are explained in the palette on the left. The scale bar shows ten times the average standard error (s.e.) of the entries in the sample covariance matrix. Drift parameter is shown on the x-axis.

**Figure 4:**
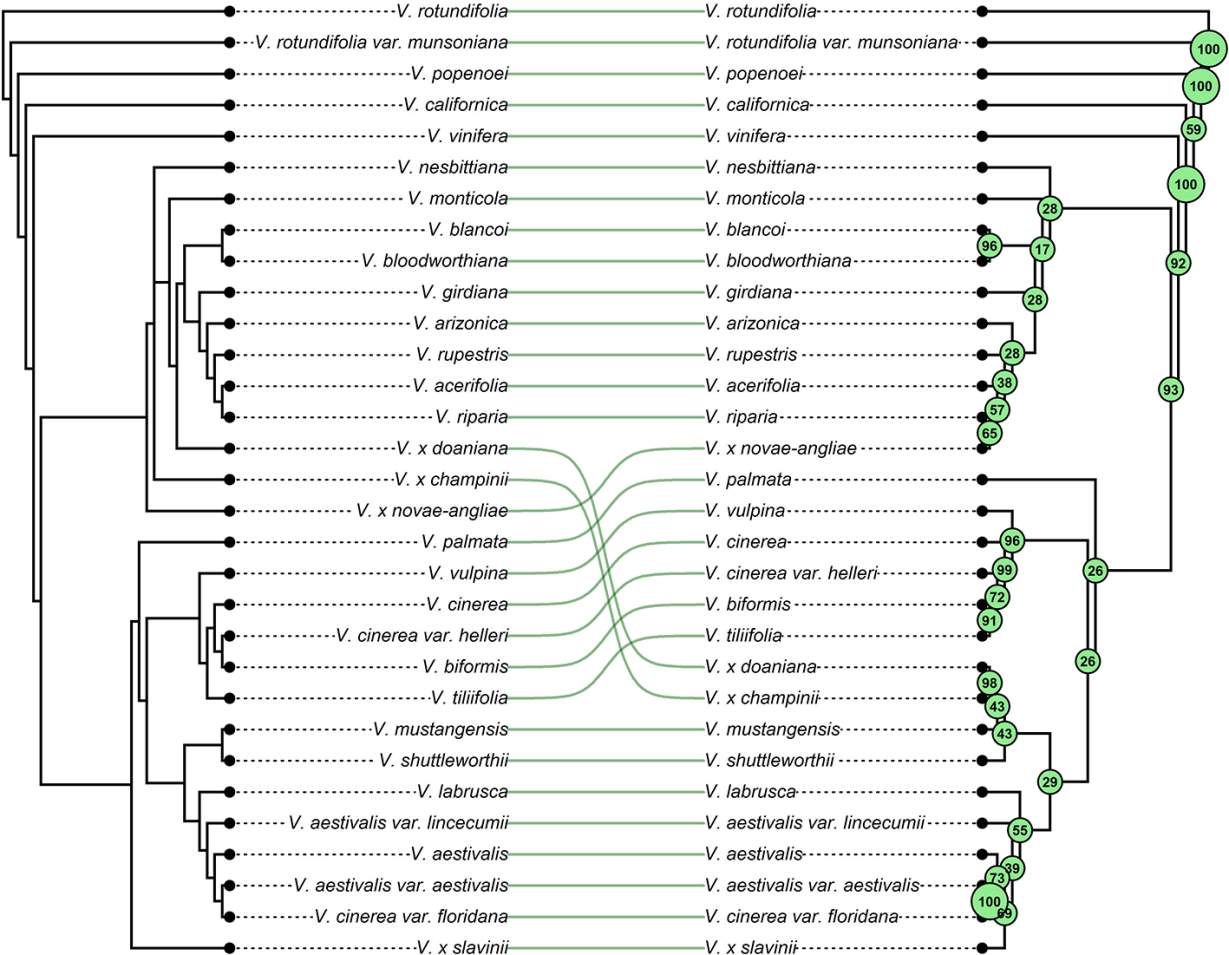
The figure shows the phylogenetic relationships of grapes referred to this work. Linking lines between corresponding tips are used to highlight changes in tree topology and taxa placements due to the addition of migration events. Left: the strict consensus tree between the two the best-scoring Maximum Likelihood (ML) tree topology obtained from 400 ML searches performed by TreeMix under the strictly bifurcating model. Right: strict consensus tree of the forty ML trees inferred by TreeMix allowing nine migration events. Bootstrap values (500 replicates) are shown for each node.

The final data set included 459 accessions from 31 *Vitis* species and 3,885 SNPs after data cleaning and sample selection (**Supplementary file S2**). The 90 American accessions removed from the analysis and reasons for exclusion are shown in **Supplementary Table S2** and **Supplementary file S2**. Our results indicated a rapid LD decay in all examined species (**Figure 1A** and **Supplementary Figure S1**). Based on these results we chose a distance of 200 kb, which greatly exceeds the extent of the observed LD, as a reference distance threshold and, accordingly, we identified 20 SNPs as the appropriate block size to account for most of the LD present among loci (**Figure 1B**). TreeMix found 99 best-scoring ML trees [ln(likelihood) = 837.613] out of the 400 ML searches performed. All ML trees were similar in terms of topology and branch lengths, converging toward only two nonidentical but very close solutions (R–F distance = 2). ML trees (**Figure 2A** and **Supplementary Figure S2**) explained 91.7% of the variance in relatedness between taxa thus justifying the assumption underlying the model implemented in TreeMix that the history of sampled taxa is approximately tree-like. On the other hand, residuals from the fit of the model to the data showed that the tree could not completely explain the ancestry of a number of species, among which the naturally occurring hybrids stood out (**Figure 2B**). All final runs with eight migration events reached the same ML value [ln(likelihood) = 2691.54] and explained 98.6% of the variance in relatedness between taxa, showing a substantial improvement over the tree without migrations (**Figure 3** and **Supplementary Figure S3**). Results are summarized in **Table 1**, with migration edges labelled with capital letters from A to H. Details of each run are given in **Supplementary Table S3** and **Table S4A–H**. Remarkably, no migration event involved *V. vinifera* and inferred migrations edges remained stable across different runs. Only 2 slightly different topologies (R-F distance = 2) were found in the final ML trees. Unresolved nodes and changes in the tree topology and taxa placement caused by the addition of migration events are shown in **Figure 4**.

**Table 1:** Migration edges inferred by TreeMix during the Maximum Likelihood (ML) searches carried out fitting eight gene flow events.

Migration edge	Origin	Destination	N	$\bar{w}$	${\bar{w}}_{j}$	${\bar{SE}}_{j}$	p-value	MS	MS_E_
**A**	(*V. labrusca*)	(*V.* x *novae-angliae*)	40	0.492	0.492	0.020	< < 1.000 E^-10^	69	–
**B**	(*V.* x *novae-angliae*)	(*V.* x *slavinii*)	40	0.444	0.444	0.025	< < 1.000 E^-10^	63	–
**C**	*Euvitis* group	(*V.* x *champinii*, *V.* x *doaniana*, *V. mustangensis*, *V. shuttleworthii*)	40	0.154	0.154	0.064	0.008	26	62 ^§^
**D**	(*V. acerifolia*, *V. arizonica*, *V. blancoi*, *V. bloodworthiana*, *V. girdiana*, *V.* x *novae-angliae*, *V. riparia*, *V. rupestris*)	(*V. tiliifolia*)	40	0.431	0.431	0.025	< < 1.000 E^-10^	16	23 ^#^
**E**	(*V.girdiana*)	(*V. californica*)	40	0.347	0.347	0.055	1.333 E^-10^	74	–
**F**	(*V. rupestris*)	(*V.* x *champinii*, *V.*x *doaniana*)	40	0.488	0.487	0.018	< < 1.000 E^-10^	35	36 ^*^
**G**	(*V. acerifolia*)	(*V.* x *doaniana*)	40	0.358	0.358	0.023	< < 1.000 E^-10^	32	–
**H**	(*V. californica*)	(*V. bloodworthiana*)	40	0.140	0.140	0.035	3.843 E^-05^	10	–

Migration edges are labelled with capital letters from A to H. For each migration the number of independent runs (N) used to calculate mean values, the mean weight on the edge ($\bar{w}$), the jackknife estimate of the weight averaged over the N independent runs (${\bar{w}}_{j}$) and the jackknife estimate of the standard error averaged over the N independent runs (${\bar{SE}}_{j}$) are shown in the table. The least significant p-value (p-value) recovered by TreeMix during the independent runs is also shown. Overall, results were found to be very stable across different runs (see **Supplementary Tables S3** and **S4**). Origin, the set of species found in the subtree below the origin of the migration edge; Destination, the set of species found in the subtree below the destination of the migration edge; MS, migration support; MS_E_, extended migration support. Both MS and MS_E_ are defined in **Supplementary file S1**. §, MS_E_ value is computed considering as valid destination of migration the set of species shown in the column ‘Destination’ as well as any of its proper subsets with at least *2* elements. #, MS_E_ value is computed considering as valid origin of migration the set of *n* species specified in the column ‘Origin’ as well as its proper subsets with at least *n*-3 elements. *, MS_E_ value is computed considering as valid origin of migration the set of species shown in the column ‘Destination’ as well as any of its proper subsets.

### *f*3 and *f*4 Statistics

#### Series *Ripariae*

TreeMix analysis identified the species placed in the series *Ripariae* as likely parent species of all naturally occurring hybrids included in this work (**Figures 3** and **4**). Thus, before investigating the relationships between the series *Ripariae* and hybrids, we first tested whether gene flow existed between *V. acerifolia*, *V. riparia,* and *V. rupestris*. Using the *f*3 test, we found no evidence of admixture between these three species (**Table 2**). We further examined their relationships using the *f*4-statistic of treeness and including *V. arizonica* Munson as fourth taxon. The tree [(*V. acerifolia*, *V. riparia*), (*V. arizonica*, *V. rupestris*)], compatible with TreeMix results, passed the test, while the two alternative trees connecting the four taxa failed the *f*4 test (**Table 3**). These results indicated that the first topology was consistent with the data without evidence of admixture events.

**Table 2:** Admixture *f*3 statistic and ‘outgroup’ *f*3 statistic.

*Hp*	*Admixture f3*(*X; Y, W*)						
	*X*	*Y*	*W*	Z-score	p-value		H-B
**Series *Ripariae***	*V. acerifolia*	*V. riparia*	*V. rupestris*	3.29	n.s.		n.s.
	*V. riparia*	*V. acerifolia*	*V. rupestris*	5.31	n.s.		n.s.
	*V. rupestris*	*V. acerifolia*	*V. riparia*	6.25	n.s.		n.s.
**Migration A**	***V.* x *novae-angliae***	*V. acerifolia*	*V. labrusca*	−17.11	6.25 x 10^-66^		****
			*V. aestivalis* var. *lincecumii*	0.90	n.s.		n.s.
**Migration B**	***V.* x *slavinii***	*V. shuttleworthii*	*V. riparia*	−17.03	2.4 x 10^-65^		****
			*V.* x *novae-angliae*	−10.31	3.3 x 10^-25^		****
**Migration D**	*V. tiliifolia*	*V. biformis*	*V. monticola*	−13.55	4.0 x 10^-42^		****
***Hp***	***Outgroup f3*(*O; T, R_i_*)**						
	***O***	***T***	***R***	***f3***	**Z-score**	**p-value (|Z_diff_|)**	**H-B**
**Migration B**	*V. aestivalis* var. *lincecumii*	***V.* x *slavinii***	*V. riparia*	0.01487	10.18	4.5 x10^-4^	**
*V. aestivalis* var. *lincecumii*	***V.* x *slavinii***	*V.* x *novae-angliae*	0.00789	6.67			

Tested hypotheses are given in the first column (Hp). Migrations A–H refer to migration edges shown in **Table 1**. When the parentage of a natural occurring hybrid is investigated, the name of the hybrid species is highlighted in bold. Z-scores computed using a block jackknifing procedure and their relative p-values are reported for each test. Statistical significance was further assessed according to Holm-Bonferroni (H-B) correction for multiple testing (n = 9). The interpretation of these tests is described in Materials and Methods. |Z_diff_|: the absolute value of the difference between the Z-scores obtained testing alternative hypotheses in the outgroup f_3_ test; n.s., not significant; **, p < 0.01; ****, p<< 0.0001.

**Table 3:** Test for treeness: *f*4 statistic.

*Hp*	*f4* (*X, Y; W, Z*)						
	*X*	*Y*	*W*	*Z*	Z-score	p-value	H-B
**Series Ripariae**	*V. acerifolia*	*V. riparia*	*V. arizonica*	*V. rupestris*	−1.21	n.s.	n.s.
	*V. acerifolia*	*V. arizonica*	*V. rupestris*	*V. riparia*	−2.93	3.4 x 10^-03^	*
	*V. acerifolia*	*V. rupestris*	*V. arizonica*	*V. riparia*	−3.40	6.7 x 10^-04^	**
**Migration B**	***V.* x *slavinii***	*V. aestivalis* var. *lincecumii*	*V.girdiana*	*V. riparia*	−6.73	1.7 x 10^-11^	****
				*V.* x *novae-angliae*	−0.35	n.s.	n.s.
**Migration C**	*V. vulpina*	*V. mustangensis*	*V. popenoei*	*V. californica*	−5.33	9.8 x 10^-8^	****
		*V. shuttleworthii*			−3.85	1.2 x 10^-4^	**
		*V. mustangensis*	*V. nesbittiana*		0.03	n.s.	n.s.
		*V. shuttleworthii*			−0.77	n.s.	n.s.
**Migration E**	*V. popenoei*	*V. californica*	*V.girdiana*	*V. blancoi*	−3.95	7.8 x 10^-05^	**
			*V. arizonica*		1.35	n.s.	n.s.
**Migration F & Migration G**	*V. shuttleworthii*	***V. x champinii***	*V.girdiana*	*V. rupestris*	3.11	1.87 x 10^-3^	*
				*V. arizonica*	1.36	n.s.	n.s.
		***V.* x *doaniana***		*V. rupestris*	3.17	1.5 x 10^-3^	*
				*V. arizonica*	1.85	n.s.	n.s.
				*V. acerifolia*	5.63	1.8 x 10^-8^	****
**Migration H**	*V. popenoei*	*V. californica*	*V. biformis*	*V. bloodworthiana*	2.10	0.035	n.s.
				*V. monticola*	1.86	n.s.	n.s.

Tested hypotheses are given in the first column (*Hp*). Migrations A–H refer to migration edges shown in **Table 1**. When the parentage of a natural occurring hybrid is investigated, the name of the hybrid species is highlighted in bold. Z-scores computed using a block jackknifing procedure and their relative p-values are reported for each test. Statistical significance was further assessed according to Holm-Bonferroni (H-B) correction for multiple testing (n = 18). The interpretation of *f*4 test is described in Materials and Methods. n.s., not significant. *, p < 0.05; **, p < 0.01; ****, p<< 0.0001.

### Natural Hybrids

All final ML trees obtained fitting eight migrations placed the naturally occurring hybrid *V.* x *novae-angliae* as the sister to *V. riparia,* supporting a close relationship between these two species (**Figures 3** and **4**). Migration edge A (*w* = 49.2%) identified *V. labrusca* as the second parent species of *V.* x *novae-angliae* (**Table 1** and **Figure 3**). This finding implies that allele frequencies in *V. labrusca* are more similar to those in *V.* x *novae-angliae* than would be expected from their position in the tree (**Figures 3** and **4**). To confirm this expectation, we used the *f*3-statistic in the following form: *f*3(*V.* x *novae-angliae*; *V. acerifolia*, *W*), where *W* was either *V. labrusca* or *V. aestivalis* var. *lincecumii* (Buckley) Munson. The *f*3-statistic showed strong evidence of admixture when *V. labrusca* was used as taxon *W*. Replacing *V. labrusca* with *V. aestivalis* var. *lincecumii* led instead to nonsignificant results (**Table 2**). Thus, our results supported the conclusion that *V.* x *novae-angliae* is the result of at least one admixture event involving *V. labrusca* and *V. riparia*.

The position of *V.* x *slavinii* in the ML trees suggested that *V.* x *slavinii* derives part of its ancestry from the clade of the “*V. aestivalis*-like” species (**Figure 3** and **Figure 4**), while migration edge B (*w* = 44.4%) indicated that the remaining part of its ancestry derives from *V.* x *novae-angliae* (**Table 1** and **Figure 3**). Since *V.* x *novae-angliae* is related to *V. riparia*, one of the putative parent species of *V.* x *slavinii*, we decided to investigate further this point. We applied the *f*3-statistics in the form: *f*3(*V.* x *slavinii*; *V. shuttleworthii*, *W*), where *W* was either *V. riparia* or *V.* x *novae-angliae*. Both tests were rejected with highly significant results, confirming the presence of admixture (**Table 2**). Then, we turned to a more model-based test, by calculating the following configurations of the *f*4 test: *f*4(*V.* x *slavinii*, *V. aestivalis* var. *lincecumii*; *V.girdiana*, *Z*), where *Z* was either *V. riparia* or *V.* x *novae-angliae*. *V.* x *slavinii* showed a significant signal of admixture only when *V. riparia* was included in the test (**Table 3**), suggesting a stronger relation with this species than with *V.* x *novae-angliae*. To confirm this, we computed the ‘outgroup’ statistic *f*3(*V. aestivalis* var. *lincecumii*; *V.* x *slavinii*, *R*), where *R* was either *V.* x *novae-angliae* or *V. riparia*. According to these tests, *V.* x *slavinii* was found to be more closely related to *V. riparia* than to *V.* x *novae-angliae* (|Z_diff_| score ~ 3.5; see **Table 2**).

*V. mustangensis* was sister to the clade composed of *V.* x *doaniana* and *V.* x *champinii* in all final ML solutions, showing that *V. mustangensis* has been involved in the ancestry of *V.* x *doaniana* and *V.* x *champinii* (**Figures 3** and **4**). Migration edges F (*w* = 48.8%) and G (*w* = 35.8%) indicated the involvement of *V. acerifolia* and *V. rupestris* in the parentage of *V.* x *doaniana* and *V.* x *champinii*. We investigated the ancestry of these two natural hybrids by calculating the following configurations of the *f*4 test: *f*4(*V. shuttleworthii*, *Y*; *V.girdiana*, *Z*), where *Y* was either *V.* x *doaniana* or *V.* x *champinii* and *Z* was one of *V. acerifolia*, *V. rupestris,* and *V. arizonica*. While trees including *V. arizonica* passed the test, any configuration including *V.* x *doaniana* or *V.* x *champinii* was rejected (**Table 3**). These results supported the contribution of *V. acerifolia* and *V. rupestris* to the ancestry of *V.* x *doaniana* and the contribution of *V. rupestris* to the ancestry of *V.* x *champinii*.

### Detecting Additional Evidence of Reticulate Evolution

TreeMix inferred a likely ancient genetic contribution to the ancestry of the clade composed of *V.* x *doaniana*, *V.* x *champinii, V. mustangensis,* and *V. shuttleworthii* House (migration C: *w* = 15.4%; **Table 1** and **Figure 3**). To verify this result, we focused our attention on *V. mustangensis* and *V. shuttleworthii.* We computed the test *f*4(*V. vulpina*, Y; *V. popenoei*, *V. californica*), where Y was one of *V. mustangensis* and *V. shuttleworthii*. Both configurations failed the *f*4 test, while after the replacement of *V. popenoei* with *V. nesbittiana* Comeaux both tests were passed, confirming the existence of this ancient introgression (**Table 3**).

Migration edge D (*w* *=* 43.1%), predicts that allele frequencies in *V. tiliifolia* Humb. & Bonpl. ex Willd are closer to those in *V. monticola* than would be expected based on the inferred tree (**Table 1** and **Figure 3**). This expectancy was confirmed by computing the test *f*3(*V. tiliifolia*; *V. biformis*, *V. monticola*), which as expected, rejected the null hypothesis of treeness (**Table 2**).

Migration edge E (*w* = 34.7%) disclosed the existence of gene flow between *V.girdiana* and *V. californica* Benth. (**Table 1** and **Figure 3**). We verified this signal computing the test *f*4(*V. popenoei*, *V. californica*; *V.girdiana*, *V. blancoi*) which rejected the hypothesis of no gene flow, while substituting *V.girdiana* with *V. arizonica* that was not predicted to be involved in the admixture event resulted in a tree that passed the *f*4 test (**Table 3**).

Migration edge H (*w* = 14.0%) indicates allele frequencies in *V. californica* are more related to those in *V. bloodworthiana* than would be expected from the inferred phylogeny (**Table 1** and **Figure 3**). We verified this using the following test of treeness: *f*4(*V. arizonica*, *V.girdiana*; *V. aestivalis* var. *lincecumii*, *V*. *cinerea* var. *floridana*). As predicted by TreeMix the proposed tree failed the *f*4 test, while substituting *V. aestivalis* var. *lincecumii* with *V. labrusca* resulted in a tree that passed this test (**Table 3**). We sought to confirm the signal of gene flow upheld by migration edge H calculating the *f*4 statistics: *f*4(*V. popenoei*, *V. californica*; *V. biformis*, *V. bloodworthiana*). As predicted by TreeMix the proposed tree failed the *f*4 test, even if with a level of significance lower than in other admixture events, while substituting *V. bloodworthiana* with *V. monticola* resulted in a tree that passed this test (**Table 3**).

Statistical significance of migrations was further assessed according to Holm-Bonferroni method to control the familywise error rate for multiple *f*3 and *f*4 tests. After applying the Holm-Bonferroni correction all migrations were still statistically significant, with the only exception of migration edge H (**Table 3**).

## Discussion

### Natural Hybrids

Nowadays, natural hybridization is a recognized aspect of American grapes evolution. However, uncertainty still remains relative to the species involved in the ancestry of the named hybrid taxa native of North America. Here, new insights gained based on genomic data, have enhanced our understanding of the origin of the natural hybrids proposed in literature.

#### *V.* x *champinii* and *V.* x *doaniana*

*V.* x *champinii* is distributed through the Edwards Plateau (Texas) and used to produce rootstock and cultivars due to its drought tolerance and capacity to grow on calcareous soil (Munson, 1909; Comeaux, 1987; Moore, 1991; Comeaux, 1991). The origin of this species is disputed and several hypotheses have been proposed. Bailey (1934) and Moore (1991) suggested that it is produced by the spontaneous cross between *V. mustangensis* and *V. rupestris*, but other putative parents, such as *V. cinerea* var. *helleri* and *V. monticola*, have been proposed (Heinitz et al., 2019). Zecca et al. (2019), based on plastome data, suggested a possible *V. rupestris*-like origin of *V.* x *champinii* maternal lineage. On the contrary, Pinto-Maglio et al. (2010) showing a different number of submetacentric chromosomes between *V.* x *champinii* and other species, including *V. rupestris*, *V. cinerea* (Engelm.) Millardet, *V. labrusca* and *V. rotundifolia*, stated that none of the grapes analyzed could be a parent of *V.* x *champinii*.

*V.* x *doaniana* is spread over a restricted area between central Texas and Oklahoma. Bailey (1934) and Moore (1991) observed and described several samples, defining the boundaries and the traits useful to distinguish *V.* x *doaniana* from other species, like *V. arizonica* Engelm. and *V.* x *champinii* growing in the same area. Both authors agreed defining the species as derived from a cross between *V. mustangensis* and *V. acerifolia*. Recently, Péros et al. (2011) observed samples of *V.* x *doaniana* clustered together with species of series *Ripariae* in a phylogenetic tree based on plastid data and they suggested that the maternal parent of *V.* x *doaniana* could be found within the series *Ripariae*.

Our findings strongly corroborated the hybrid origin of *V.* x *champinii* and *V.* x *doaniana,* indicating *V. mustangensis* as one of their likely parent species (**Figure 3**). Moreover, our results indicated *V. rupestris* and *V. acerifolia* as the second parent species of *V.* x *champinii* and *V.* x *doaniana*, respectively (**Figure 3**, **Tables 1** and **3**). TreeMix inferred a single migration edge from *V. rupestris* to the most recent common ancestor (MRCA) of *V.* x *doaniana* and *V.* x *champinii* (migration edge F, **Figure 3** and **Table 1**). A second migration edge was inferred from *V. acerifolia* to the tip representing *V.* x *doaniana* (migration edge G: **Figure 3** and **Table 1**). The *f*4 statistic upheld TreeMix results supporting both migration events (**Table 3**). Overall, our results supported the origin of *V.* x *champinii* as the result of a spontaneous cross between *V. mustangensis* and *V. rupestris* (ca. 49% of *V.* x *champinii* ancestry). On the other hand, they would seem to suggest a different complex scenario for the origin of *V.* x *doaniana*, involving migration events from both lineages of *V. acerifolia* and *V. rupestris*.

#### *V.* x *novae-angliae*

*V.* x *novae-angliae*, commonly named the pilgrim grape, is diffused in the states of New England (Fernald, 1917). Fernald (1917) after describing several typical and fixed traits, claimed that the pilgrim grape was recognized as having *the official status* of *an independent species.* In contrast, Bailey (1934) described *V.* x *novae-angliae* as a high and vigorous hybrid climber with intermediate characters between *V. labrusca* and *V. vulpina* auct. non L. Despite the different views about the origin of the pilgrim grape, both authors agreed that its appearance occurred in a distant past. Moore (1991), after extensive observations of Fernald’s specimens, established the hybrid origin of *V.* x *novae-angliae* and proposed *V. labrusca* and *V. riparia* as putative parents. The Moore’s hypothesis was also supported by some spontaneous hybrids that had been collected in the states of New York and Tennessee where the ranges of both putative parents overlap (Hedrick, 1908). Furthermore, hybridization among *V. labrusca*, *V. riparia,* and *V. vinifera* has been well documented in viticulture and several hybrids with *labrusca*-*riparia* parentage, characterized by a typical foxy aroma, have been derived since from the 19^th^ century (Millardet, 1880). These hybrids were cultivated extensively also in Europe until the early decades of the 20^th^ century, when they were outlawed. Even today there are still traces of their re-naturalization in several European countries (Ardenghi et al., 2015).

Our results clearly identified *V. labrusca* and *V. riparia* as the two parent species of *V.* x *novae-angliae*, confirming Moore’s hypothesis (migration edge A: **Figure 3**, **Tables 1** and **2**). The estimated admixture proportions close to fifty percent (with ca. 49% of the ancestry coming from *V. labrusca*, **Table 1**).

#### *V.* x *slavinii*

*V.* x *slavinii*, was originally classified as a species in its own right with traits that resemble those of *V. argentifolia* Munson and *V. vulpina* auct. non L (Bailey, 1934). A few specimens were collected on the banks of the Genesee River near Rochester in New York State, but in general, little information is available on this taxon. *V.* x *slavinii* was not included in the Flora of North America North of Mexico (Moore and Wen, 2016), but it is currently described in the GRIN-Taxonomy database (USDA, 2019) as a possible hybrid between *V. aestivalis* and *V. riparia*. It is known to possess a moderate resistance to the dagger nematode (*Xiphinema index*) which is why it has been used in breeding rootstocks resistant to this ectoparasite (Boyden and Cousins, 2003).

TreeMix analyses identified an admixture event from the *V.* x *novae-angliae* into the *V.* x *slavinii* (migration edge B: **Figure 3**, **Table 1**) that account for about 44% of *V.* x *slavinii* ancestry. This is not particularly surprising if we consider that *V. riparia* is one of the parent species of *V.* x *novae-angliae*. In order to disentangle this point we applied the *f*3 and *f*4 statistics including *V. riparia* as possible parent species. Overall the *f*3 statistic, the *f*4 statistic and the ‘outgroup’ *f*3 statistic yielded evidence in favour of *V. riparia* over *V.* x *novae-angliae* as parent species of *V.* x *slavinii* (**Tables 2** and **3**). Therefore, we interpreted the global solution adopted by TreeMix as a compromise obtained by the model in an attempt to explain the origin of two hybrids that share a substantial part of their ancestry, while fitting other extensive gene flows at the same time. The position of *V.* x *slavinii* in the final ML trees was found to be stable across different runs and when branch lengths are taken into account the specie appeared closer to *V. aestivalis* than to other taxa of the “*V. aestivalis*-like” clade (**Figure 3**). As proposed by the GRIN-Taxonomy database, the evidence we found points to a hybrid origin of *V.* x *slavinii* that involve in its parentage *V. riparia* and, most likely, *V. aestivalis*.

As also shown by our work, the formation of natural hybrids in American grapes is a spontaneous evolutionary process. It is important to note that natural hybrids recognized as species deserve a particular level of protection (Allendorf et al., 2001). Despite some hybrid grapes being preserved in ex-situ collections because recognized as important economic resources, their actual status of conservation in nature should be considered critical and thus carefully monitored. For example, very few information is available on the conservation status of *V.* x *slavinii* while several factors driven by human activities (e.g. urbanization, agriculture and grazing) are contributing to the reduction of the areal distribution of *V. x champinii* and *V. x doaniana* with severe demographic and genetic consequences (Comeaux et al., 1987; Comeaux, 1991; Heinitz et al., 2019).

Finally, particular caution must be exercised concerning the hybridizations that involved *V. x novae-angliae* and *V. x slavinii*. Our results are based on a limited number of samples thus we suggest that the interpretation of migrations that involve these species should be circumscribed to samples included in our dataset. We cannot ruled out *a priori* that these two species may exhibit a more complex genetic structure or a wider genetic variability than observed in this study.

### Additional Evidence of Reticulate Evolution in American Wild Grapes

In addition to migration related to the natural hybrid species described in literature, our analysis also pointed out a number of gene flows involving other species. At first glance, this could be surprising because according to some authors the reproductive isolation in modern grapes would be held by differences in spatial distribution, different times of flowering and the aptitude to grow on different soils (Comeaux et al., 1987; Callen et al., 2016). Nonetheless, the idea of a complex reticulate evolution within the *Vitis* genus has recently been put forward by several authors (Péros et al., 2011; Zecca et al., 2012; Aradhya et al., 2013; Wan et al., 2013; Miller et al., 2013; Goto-Yamamoto et al., 2015; Moore and Wen, 2016; Ma et al., 2018b). Ancient grapes may have had spatial distributions and biological features unknown nowadays, and therefore we cannot exclude that under certain circumstances they may have been driven to overlap and crossbreed. Admixture events, occurred in a distant or recent past, may have left detectable genomic footprints observable today in modern grapes.

The gene flow, corresponding to migration edge C (**Figure 3** and **Table 1**), seemed to trace back to an ancient form of *Vitis*, most probabily an ancestor of the current subgenus *Vitis* subsequent to the divergence of the two subgenera. Today we know that *Vitis* x *Muscadinia* hybrids are rare and normally sterile as a result of having an odd number of chromosomes (Hickey et al., 2019), but still linkage map analysis indicates an overall high degree of collinearity between the physical maps of the two subgenera (Lewter et al., 2019). It is likely that the ancient grapes of subgenus *Vitis* were more similar to the muscadines than modern grapes (Lewter et al., 2019), which could explain the origin of the migration shift toward the modern *Muscadinia*.

Migration D indicated that *V. tiliifolia* has also experienced a complex evolutionary history showing evidence of an considerable introgession (ca. 43% of its ancestry) from an ancestor of current species (**Figure 3** and **Table 1**). Due to the wide distributional range of *V. tiliifolia* further studies will need to be undertaken to provide a proper interpretation of this admixture event.

Another gene flow of particular interest identified during our work was the one involving two species typical of the West Coast: *V. californica*, endemic to California and Oregon, and *V. girdiana*, endemic to California and Mexico (Baja California) (migration edge E, **Figure 3** and **Table 1**). These two endemic species, in addition to showing evolutionary differences in the plastomes (Wen et al., 2018), differ in well-defined traits such as leaf shape and tomentum, berry size and seed morphology. On the other hand, their flowering times partially overlap and they have similar habitat preferences (Wada and Walker, 2012; Moore and Wen, 2016). Although *V. californica* has a northern distribution and *V. girdiana* is spread southward, the two species show a contact zone in central California (Olmo and Koyama, 1980; Moore and Wen, 2016). California is characterized by high plant diversity (Loarie et al., 2008) and several authors have shown that past climate changes have promoted hybridization within different genus, such as *Quercus* (Ortego et al., 2014), *Brassica* (Chunco, 2014) and *Rhus* (Young, 1974). Ravaz (1902) proposed a hybrid origin of *V. girdiana* indicating *V. californica* and *V. vinifera* as putative parents. On the contrary, Munson (1909) acknowledged *V. girdiana* and *V. californica* as two independent species, native of North America, excluding *V. vinifera* from their original ancestry. However, the grapevine has been cultivated in California since 1769 and several authors have put forward the hypothesis that the increase in viticulture during last century may have caused the involuntary transfer of domestic alleles into wild populations (Wagner, 1974; Moore and Wen, 2016). Hybridization or introgression caused by to contact with nonnative taxa is damaging because several parts of the genome can be afflicted, increasing the probability of extinction especially in small or periphery populations (Mallet, 2005; Todesco et al., 2016). In a recent molecular study based on a large sampling, Dangl et al. (2015) have confirmed the independent origin of *V. californica* and *V. girdiana* but have also shown evidences of introgression from grapevine cultivars, claiming the danger of genomic swamping.

Our results recognized the independent origin of both species, but also revealed that *V. californica* traces a considerable fraction of its ancestry (ca. 35%) to *V. girdiana*, confirming without doubt that the two species crossbred. During our sample selection processes we have discarded two accessions of *V. californica* and one accession of *V. girdiana* because of their hybrid origin (**Supplementary Table S2**). In particular the two samples of *V. californica* (DVIT 1361 and DVIT 1836) were first identified in this study by the RaxML analysis as possible hybrids with ancestry from *V. vinifera*. The exclusion of these three hybrids has prevented TreeMix from identifying gene flow from *V. vinifera* to *V. californica* and *V. girdiana*, but it has allowed the risk of overestimation of the introgression from *V. girdiana* into *V. californica* to be avoided, leaving us confident that the observed effect is the genuine result of natural processes.

Migration edge H, involving *V. californica* and *V. bloodworthiana*, was the last significant admixture event identified by TreeMix. Our results seem to indicate that the Mexican *V. bloodworthiana*, now growing at high elevations in the Sierra Madre Occidental in the states of Sinaloa and Durango (Comeaux, 1991), have experienced an introgression from *V. californica* during its evolutionary history (ca. 14%, **Tables 1** and **3**). We speculate that this introgression might have due to contacts following postglacial range expansion (see next paragraph). However, because this migration was rejected after the Holm-Bonferroni correction for multiple *f*4 tests (**Table 3**), we recommend that further research is undertaken to estimate the strength of this gene flow.

### Climate Changes, Geographical Constraints, and Reticulate Evolution in American Grapes

Our results have shown that reticulate evolution has played an important role in shaping the existing diversity of American grapes. It is worth noting that most of the observed gene flows have involved taxa currently spread through the southern regions of North America. It is well-known past climate changes have influenced the distribution of animals and plants in North America (Hewitt, 1996) and it has been widely demonstrated that the breakdown of spatial barriers may be triggered by climate changes (Chunco, 2014). Although our analysis did not allow us to estimate the time of admixture events we can easily assume that even grape vicissitudes were affected by past glacial cycles. Under glacial conditions numerous plant species were driven to where appropriate conditions continued to subsist, finding shield in restricted enclaves diffused mainly in Texas, California, Florida or Mexico (McLachlan et al., 2005; Swenson and Howard, 2005). It is likely that grapes also suffered the same fate. During these southward migrations, American grapes could have experienced a drastic range contraction and habitat fragmentation as occurred for several lowland taxa (Folk et al., 2018). Moreover, the funnel-shaped southern North America and the North-South direction of the main mountain ridges may have channelled these migrations to the same suitable southern regions, forcing the sympatry of many *Vitis* species (Zecca et al., 2012). The south-eastern part of North America has always been characterized by a high number of coexisting grape species, as evidenced by fossil records and by the number of species living there today (Tiffney, 1994; Gong et al., 2010; Weems et al., 2017; Heinitz et al., 2019), offering the ideal context for hybridization between different taxa. Moreover, Texas have offered unique characteristics among regions of North America, because of its great variability in climate and habitat. In particular, the Edwards Plateau located in central Texas has offered a cool and moist pluvial climate rich in forest and characterized by the presence of several endemic and hybrid taxa (Remington, 1968; Delcourt and Delcourt, 1993). The grapes would have found an ideal habitat to grow and the forced coexistence of interfertile taxa could have promoted local hybridization events (Loehle, 2007). Admixed individuals may have acquired new capabilities to cope with the evolving environmental conditions, increasing their competitiveness toward other conspecific individuals or even toward other species, thus allowing the genomic signature of these events to be preserved over time. Hybridization events may also have occurred due to contacts following postglacial range expansion. Several secondary contact regions have been recognized in southern North America. Alabama and adjacent states along the Coastal Plain have been proposed as zones of hybridization for several species that arose from the refugia in Texas and Florida (Remington, 1968; Swenson and Howard, 2005). During the Holocene, hybridization within secondary contact regions has been detected for many taxa along the Western Cordillera, particularly in some Mexican regions (e.g. Durango, Oaxaca, Chiapas, and Veracruz) and in California (Remington, 1968; De Castro et al., 2013; Ortego et al., 2014; Ávila-Flores et al., 2016; Baena-Díaz et al., 2018), right where *V. bloodworthiana*, *V. tiliifolia,* and *V. girdiana*, *V. californica* are spread. Thus, even the range expansion which occurred during interglacial phases may have favoured admixture events among grapes.

### Phylogenetic Implications

Hybrids are traditionally considered taxa that possess intermediate characters between their parents and are commonly expected to cluster together with one of their parent species depending on with which parent they share the largest number of characters. Actually, in a phylogenetic context, this expectation may not always be fulfilled. In the heterozygous condition typical of hybrids the number of plesiomorphic characters (i.e., primitive, ancestral characters) might be close or even larger than the number of apomorphies. In fact, hybrids do not necessarily receive all apomorphic states from their parents. Therefore, it is possible for the hybrids to appear on the phylogenetic tree in an ancestral position as a consequence of this “lack of apomorphies” (Funk, 1985). From the analysis of our data using a strictly tree-like a model of evolution (**Figures 2** and **4**, left side) this is what has apparently happened. However, TreeMix has proven to be able to handle this issue effectively, grouping all hybrid taxa close to one of their parent species once gene flows between diverged species were allowed (**Figures 3** and **4**, right side). In the past putative hybrids were commonly removed from phylogenetic analysis to avoid their confounding effect, thus foregoing the consideration of an important feature of species evolution. Instead, as shown in this work, modern methods like TreeMix allow hybrids to be included in a phylogenetic framework, avoiding the loss of important information and therefore offering the potential of achieving novel insights into the history of diversification of taxa.

Our results confirmed the subdivision of genus *Vitis* into the two subgenera *Muscadinia* and *Vitis*. Within the subgenus *Vitis*, all phylogenetic trees inferred by TreeMix consistently identified two main clades whose species composition has recognized also by Wan et al. (2013). Hence, our findings supported the presence of at least two lineages from which the modern American species have radiated with the exception of only *V. californica*. The apparently surprising position of this enigmatic species is actually consistent with previous works. Several authors have reported an anomalous phylogenetic position of *V. californica* suggesting that it may possibly represent an evolutionary relict distanced from the other North American species of subgenus *Vitis* by long-term genetic separation (Péros et al., 2011; Tröndle et al., 2010; Zecca et al., 2012; Wen et al., 2018). Moore (1991) subdivided the eastern North American *Vitis* into five series based on morphological features. The addition of migration events has allowed us to recognize clades that partially resemble some of these series even if they have received low bootstrap support (**Figure 4**). The species belonging to the series *Ripariae* were all found in the same clade, clustered together with the natural hybrid *V.* x *novae-angliae* (**Figures 3** and **4**). All variants of *V. aestivalis* included in this work were always clustered in the same clade, similarly to series *Aestivales*. However, the related hybrid *V.* x *slavinii* and the species *V*. *cinerea* var. *floridana* and *V. labruscae* were also found in the same clade. While the inclusion of *V.* x *slavinii* in the group of “*V. aestivalis*-like” species is the expected outcome of the TreeMix model, the inclusion of the last two species is not so obvious. The placement of *V*. *cinerea* var. *floridana* within the “*V. aestivalis*-like” clade may perhaps indicate that the two species crossbred where their ranges overlap or, since it is known that *V. aestivalis* and *V*. *cinerea* were sometimes confused in the past (Moore and Wen, 2016), it may even indicate a case of possible misclassification.

*V. labrusca* has been included by Moore (1991) in the series *Labruscae* together with *V. mustangensis* and *V. shuttleworthii*, whereas it was considered to be a separate taxon by Planchon (1887) and Munson (1909). As recently pointed out by Wen et al. (2018) the species delimitation and the phylogenetic position of *V. labrusca* will require further evaluation. However, our results seem to support the idea put forward by these authors of a possible genetic relationship between *V. labrusca* and the *V. aestivalis* complex.

Finally, it is interesting to note that all final ML trees group together *V.* x *champinii*, *V.* x *doaniana*, *V. mustangensis,* and *V. shuttleworthii* (**Figures 3** and **4**, right side). This clade including four North American grapes correspond to the series *Coriaceae* established by Munson (1909). In their chloroplast phylogenomics of the American wild grapes Wen et al. (2018) did not recover support for this group while Péros et al. (2011), using both chloroplast and nuclear DNA found a partial support for this clade with *V. coriacea* Schuttl. ex K. Koch (= *V. shuttleworthii*) placed in an unresolved position. These conflicting results may indicate different evolutionary histories captured by nuclear and plastid DNA.

### Final Remarks on Admixture Inference

In this paper, we have presented a simple method to summarize bootstrap results providing an index of support for migration edges. This index, defined in **Supplementary file S1**, comes in two flavours the first called migration support (MS) and the second called extended migration support (MS_E_), which is just a relaxed version of the first. The utility of the extended version of this index is appreciable from the increased support provided to migration edge C (**Table 1**). We expect that the MS_E_ index will find its application especially in those migrations involving complex groups of species. Overall, the MS index has shown values that often were not in line with the p-values estimated by TreeMix for migrations (**Table 1**). These discrepancies can be explained if we bear in mind how these values were calculated. The p-values computed by TreeMix are based on a jackknife procedure which resamples subsets of available data. MS is instead a bootstrap-based index in which resampling is carried out drawing a replacement randomly from a set of data. Generally, these procedures are expected to give similar results, but it is important to note that here resampling was performed over blocks of contiguous SNPs, not over individual SNP. In this case, when gene flow is present, but the SNPs that convey the admixture signal are confined in a few blocks, the jackknife-based procedures are much more likely to detect the signal than bootstrap-based measures simply because of the way in which the resampling is carried out. This apparent shortcoming might in practice offer an interesting opportunity to capture a different kind of signal. The comparison between TreeMix results and MS index might consent the quick evaluation of whether the signature of a particular gene flow is restricted to a few blocks or instead widespread on the genome. Further studies are needed to clarify to what extent this indication is dependable.

In this paper we applied TreeMix to clarify the admixture history of the American wild grapes, including a high number of taxa. Treemix model is a simplification of the migration process and, as suggested by Pickrell and Pritchard (2012), it may have some limitations if the history of the taxa is not largely tree-like or when gene flow is not restricted to a relatively short time period (i.e., when there are repeated or prolonged hybridization events or when any combination of the two is present). Assuming a pure bifurcating model, TreeMix explained 91.7% of the variance in relatedness between the American grape taxa. Our result is not far from the case of dog breeds presented in Pickrell and Pritchard (2012) to illustrate the method. As argued by the authors if the assumption of treeness is not kept several different histories may be compatible with the data (Pickrell and Pritchard, 2012). In our analysis ML tree topologies were stable across different runs, with the only exceptions of very few nodes. More importantly, migrations edge remained exactly the same across all the 40 final runs, regardless the tree used to initialize the analysis. Therefore, even if there were different evolutionary histories compatible with the data analyzed here, their impact on our results would seem to be very limited. In our work, successive migrations coming from different species have been recognized and handled by TreeMix (*V. doaniana)*. We cannot exclude other cases in which multiple migrations or prolonged gene flow involving the same parents may have occurred. However, similar situations should be problematic when unclear results are obtained with no consistent tree structure inferred by TreeMix (Pickrell and Pritchard, 2012), which does not seem to be the case in our work.

## Conclusion

Reticulate evolution has played an important role in the diversification history of many species, especially in plants. Nonetheless, traditional phylogenetic approaches have often overlooked this aspect. American wild grapes (*Vitis*) represent an ideal model for the study of hybridization and introgression processes because natural hybrids have been proposed in literature and because several species are known to be potentially interfertile. To overcome the limitations inherent in a strictly bifurcating tree, here we have applied the TreeMix software that allowed us to infer both taxa splits and gene flows. Our results confirmed the existence of all hybrids species proposed in literature, identifying their most likely parent species. Moreover, we provide evidence of different gene flows between distantly related species. Even knowing that modern species distributions are not necessarily predictive of interactions which occurred in the past and that the behaviour of plants in the face of climate changes may be idiosyncratic (Zecca et al., 2017; Folk et al., 2018), we propose that glacial cycles can have triggered the hybridization between grapes. We discussed the phylogenetic implications of our findings showing that taking into account hybridization and introgression can provide unique insights into the evolutionary history of taxa.

The ease with which wild grapes crossbreed may be useful for breeders of the grape industry. Since rootstocks used nowadays in viticulture are the result of crosses between a limited number of accessions (Callen et al., 2016), wild species probably retain a highly unexplored potential to produce new resistant rootstocks through hybridization. On the other hand, hybridization and introgression can also have deleterious consequences on the conservation of wild species, especially when the gene flow comes from nonnative, cultivated taxa. Here, we have shown that this risk is real for American wild grapes and that adequate conservation strategies are required.

Our work represents a step forward in efforts to understand hybridization and introgression processes within New World *Vitis*. However, there are still unanswered questions that need to be addressed. For example, future work should focus on the accurate admixture time estimation and on the identification of genomic regions involved in gene flows.

## Data Availability Statement

The datasets generated for this study can be found in https://doi.org/10.6084/m9.figshare.9784325.v1.

## Author Contributions

GZ and FG designed the study and analyzed the data. GZ, FG, and ML discussed the data and wrote the manuscript.

## Conflict of Interest

The authors declare that the research was conducted in the absence of any commercial or financial relationships that could be construed as a potential conflict of interest.
