# Supplementary material for: Untangling the Evolution of American Wild Grapes: Admixed Species and How to Find Them
Source: Front Plant Sci. 2020 Feb 7;10:1814. doi: 10.3389/fpls.2019.01814 (PMC7025467; doi:10.3389/fpls.2019.01814)
Supplement: Supplementary file 1 [file DataSheet_1.zip › Supplementary material/Supplementary file S1/MSindex_User Manual v.1.01.pdf]

User Manual

MSindex.R v.1.01

(…and other stuff)

Giovanni Zecca, Massimo Labra, Fabrizio Grassi

02nd December 2019

1. Introduction

The R scripts described in this manual are designed to support the user in the TreeMix (Pickrell and Pritchard 2012) analysis pipeline described in the accompanying manuscript Zecca et al. (*Front. Plant Sci.*, 2019). This collection of scripts is not intended to be a proper R package. Rather, it is a documented set of utility functions written with our own needs in mind, using different coding styles. Scripts were mainly tested on Windows OS, so it may need to do small changes to adapt the code to a different OS.

Described functions can be used to:

- assist the choice of the SNP block size (-*k* flag) for TreeMix analysis;
- investigate TreeMix outputs;
- calculate the migration support index (MS and MS_E) from bootstrap replicates.

WE PROVIDE THIS CODE WITH NO GUARANTEES WHATSOEVER.

2. Load the scripts into the R session

For R to be able to execute these scripts, they need first to be read into memory. To do this, click the "Source R Code"/ "Source" option in RGui and RStudio or use the `source()` function to load scripts from file [type `?source()` for information].

3. Depends & Imports

Depends: ape, phytools.
Imports: tools, grDevices, maps.

4. Definitions

To compute the Migration Support (MS) index and the extended Migration Support (MS_E) index we first transform the trees inferred by TreeMix into sets whose elements are labels (names) corresponding to the tips of the original tree. Since we focus our attention on migrations, we are particularly interested in the taxa (gene pools) involved in the gene flow, rather than in the topology of the tree. Moreover, bipartition supports can already be estimated by calculating the standard

bootstrap values form the bootstrap replicates produced by TreeMix. Therefore branching pattern information is discarded during the process. The procedure is illustrated in Figure 1.

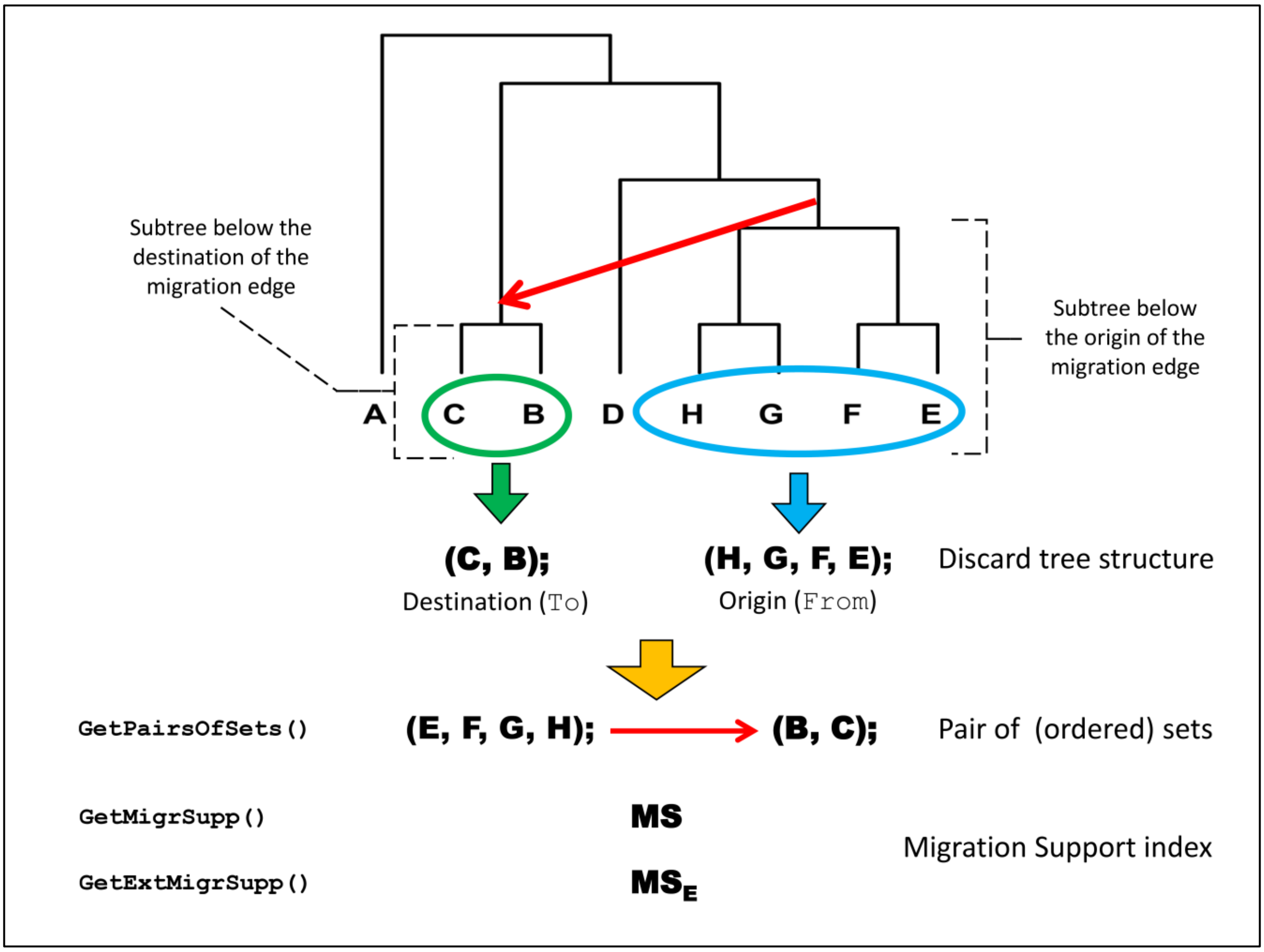

Figure 1

- **Pairs of Sets:** migration events inferred by TreeMix are converted into pairs of ordered label sets before the computation of MS index. The first set of each pair represents the subtree below the origin of the migration edge, while the second set of each pair denotes the subtree below the destination of the migration edge. Elements within each set are alphabetically ordered (Figure 1).

- **Migration Support (MS) index:** given *n* bootstrap replicates and given a pair of sets corresponding to a certain migration edge of interest, we indicate the presence/absence status of that exact pair of sets within the *i*-th bootstrap replicate with the symbol p_i. Then, the MS index is given by:

$$\mathbf{MS} = \frac{\sum_i^n p_i}{n} \cdot 100 \quad \text{where } p_i = \begin{cases} 1 & \text{if presence} \\ 0 & \text{if absence} \end{cases} \quad \text{and} \quad i = 1 \ldots n \qquad (1)$$

- **Extended Migration Support (MS$_E$) index:** given a pair of sets corresponding to a migration of interest, the MS$_E$ index is calculated accepting a user-defined relaxed matching criterion for one of the set in the pair (i.e., the "relaxed set"). When bootstrap replicates are surveyed under the relaxed criterion, positive matches occur for the entire "relaxed set" as well as for any of its proper subsets containing the minimum number of elements specified by the user. Instead, an exact-match search is performed for the other set in the pair (i.e., the "fixed set"). However, relaxing the matching criterion can sometimes result in multiple counts per replication, because different pairs of sets return a positive value (Figure 2). To prevent this we apply a simple data transformation that maps counts into presence/absence data.

Let $f\colon \mathbb{N}_0 \rightarrow \{0, 1\}$ be a function defined by $f(m) = \begin{cases} 1 & \textit{if } m \geq 1 \\ 0 & \text{if } m = 0 \end{cases}$;

given n bootstrap replicates and denoted the number of positive matches within the i-th bootstrap replicate with the symbol m_i, the MS$_E$ index is given by:

$$\mathbf{MS_E} = \frac{\sum_i^n f(m_i)}{n} \cdot 100 \quad \text{where } i = 1 \ldots n \tag{2}$$

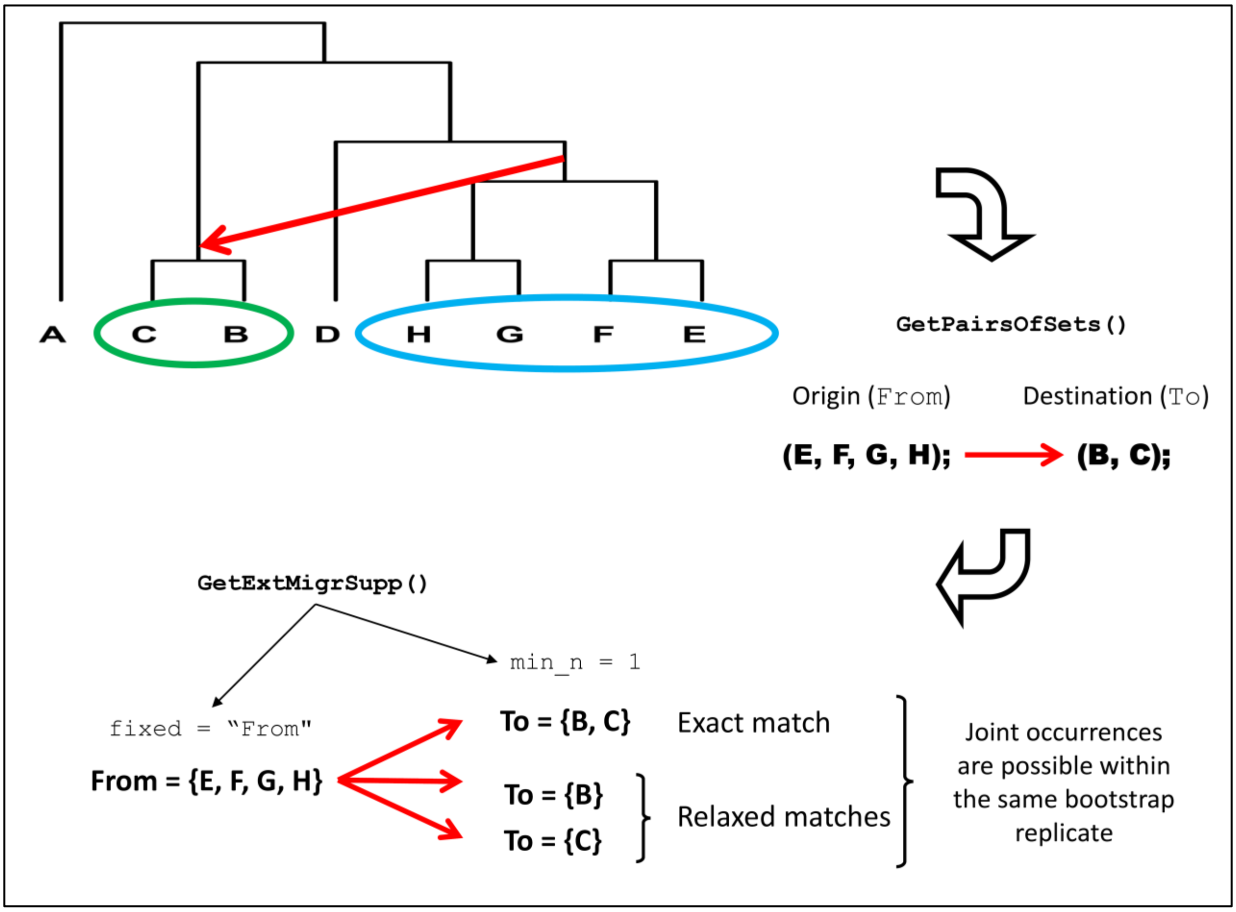

Figure 2

5. Functions

```
• MdMn_LD(inputfile=”int”, BinWidth, N, plotTitle="",
  plotMean=TRUE, colMedian = "red",colMean ="blue",xMax =
  500, refLine = 0.2, UseLastBin = FALSE)
```

Context: pairwise LD measures for multiple SNPs (r^2) have been calculated using PLINK (plink.ld file). Values can be plotted to visualize LD decay vs. inter-marker distance.

Assumptions: the input file is a standard plink.ld file with 7 columns (see PLINK manual) obtaine using the `--r2` command. This file can be selected interactively or be manually specified by the user (see below).

Arguments:

`inputfile`	character string indicating the name of the input file. If it does not contain an absolute path, the file name is relative to the current working directory. If `inputfile =”int”` (default) the input file is chosen interactively.
`BinWidth`	integer giving the number of pairwise SNP comparisons to be included in each bin. For each bin, median and (optionally) mean r^2 values are calculated from '`BinWidth`' pairwise SNP comparisons.
`N`	integer specifying the number of individuals used for LD estimation.
`plotTitle`	character string indicating the main title of the plot (Default = ""). Quotation marks are mandatory.
`plotMean`	logical; if `TRUE` (default) both the median and the mean r^2 values are plotted. If `FALSE`, only the median r^2 is plotted.
`colMedian`	character specifying the colour used to plot the median r^2 line (default: `"red"`)
`colMean`	character specifying the colour used to plot the mean r^2 line (default: `"blue"`). If `plotMean` = `FALSE`, '`colMean`' is ignored.
`xMax`	integer, a number corresponding to the maximum x-axis limit expressed in kb units (default: 500). Typically, equal to (or less than) the value applied to the `--ld-window-kb` command in PLINK (see PLINK manual).
`refLine`	double; a number between 0 and 1, depending on the organism of interest. Plot a 'reference line' to help the visual inspection of the plot (default: r^2 = 0.2). Set `refLine = NULL` or `""` to prevent its use.

`UseLastBin` logical; this option applies after that all pairwise r^2 values have been reordered by their inter-SNP distance. If `FALSE` (default), the reminder of the division (total number of pairwise r^2) /'`BinWidth`' is discarded; if `TRUE`, the reminder of that division is used to build an additional bin with width = reminder (< `BinWidth`). When the 'total number of pairwise r^2' is a multiple of '`BinWidth`', use `UseLastBin = FALSE`.

Details: this function plots the median and (optionally) the mean LD(r^2) within bins of user defined width vs. the mean inter-marker distance computed for each bin. The function assumes that LD data are in plink.ld file format.

Output: a LD decay graph vs. inter-marker distance is plotted to the active graphics window. The plot offers an overview of LD decay within the applied window.

Usage: `MdMn_LD (BinWidth = 75, N = 20, plotTitle = "My title", colMedian = "green", colMean = "yellow", refline = 0.1, UseLastBin = TRUE)`

LD decay plot titled "My title", with green (median r^2) and yellow (mean r^2) lines. Plotted values are computed using data from 20 individuals and bins of 75paiwise comparisons except for the last bin.

Hint: to plot the mean r^2 values only, set `plotMean=TRUE` and `colMedian=NULL` or`""`.

```
• mMd_LD(BinWidth, N, Names,Auto = TRUE, xMax = 500,refLine = 
  0.2,UseLastBin = FALSE)
```

Context: pairwise LD measures for multiple SNPs (r^2) have been calculated using PLINK (plink.ld file) from several species/groups. Values can be plotted in a single figure to visualize LD decay in each species, populations or chromosomes.

Assumptions: the input files are standard plink.ld files with 7 colums (see PLINK manual). Files are assumed to be in the current working directory.

Arguments:

`BinWidth` integer giving the number of pairwise SNP comparisons to be included in each bin. For each bin, median r^2 values are calculated from '`BinWidth`' pairwise SNP comparisons. The same value is applied to all input files.

`N` a character vector specifying the number of individuals used to estimate LD in each species / population / chromosome. See '`Auto`' for details.

`Names` a character vector specifying the name of each species/ population/ chromosome. See '`Auto`' for details.

`Auto` logical. If `TRUE` (default), '`Names`' is ignored (i.e., there is no need to specify any name) and a new vector '`Names`' is built automatically retrieving its elements from the names of the input files (extensions are discarded). If `Auto = FALSE`, the vector '`Names`' needs to be specified by the user.
WARNING: input files in the working directory are sorted, read and processed in alphabetical order. '`N`' and '`Names`' vectors MUST follow the same order. Type: `"list.files (path = ".", pattern = "\\.ld$")"` to check how R sorts input files.

`xMax` integer, a number corresponding to the maximum x-axis limit expressed in kb unitsb(default: 500). Typically, equal to (or less than) the value applied to the `--ld-window-kb` command in PLINK (see PLINK manual).

`refLine` double, a number between 0 and 1, depending on the organism of interest. Plot a 'reference line' to help the visual inspection of the plot (default: $r^2 = 0.2$). Set `refLine = NULL` or `""` to prevent its use.

`UseLastBin` logical; this option applies after that all pairwise r^2 values have been reordered by their inter-SNP distance. If `FALSE` (default), the reminder of the division

(total number of pairwise r^2)/'`BinWidth`' is discarded; if `TRUE`, the reminder of that division is used to build an additional bin with width = reminder (< `BinWidth`). The same choice applies to all input files. When the 'total number of pairwise r^2' is a multiple of '`BinWidth`', use the default value.

Details: this function reads multiple plink.ld files from the current working directory and it plots the median LD(r^2) computed within bins vs. the mean inter-marker distance of each bin. Data from all input files are plotted in a single panel. User must define bins width and other parameters for computation.

Output: a multiple traces LD decay graph vs. inter-marker distance is plotted to the active graphics window. The plot offers an overview of LD decay within the species/population/chromosome analysed. All LD decay traces are plotted in a single panel.

Usage:

```
mMd_LD(BinWidth = 100, N = c(15,20,18,24))
```

Multiple traces LD decay plot from four input files. Plotted values are computed using data from 15,20,18 and 24 individuals, respectively. Bin width is set to 100 pairwise comparisons. Last bin is discarded. Names are automatically retrieved from input file names.

```
mMd_LD(BinWidth = 100, N = c(15,20,18,24),Names = c('name1','name2','name3','name4'), Auto = FALSE, UseLastBin = TRUE)
```

Similar to the previous example, but here names are specified by the user and the last bin of width < 100 is not discarded.

WARNING: require "tools" package.

```
• BlockDistByChr(k, refline, int = TRUE, inputfile, colP=
  'green2',pchP = 19, colL = 'red2',typeL = 2)
```

Context: it is necessary to group together SNPs to account for linkage disequilibrium (LD) in TreeMix analysis. There is an approximate knowledge of LD decay in the organism(s) of interest, but the appropriate block size (i.e, *-k* flag) to be used in TreeMix analysis is not known.

Assumptions: The input file is a standard `plinK.map` file (see PLINK manual) including a list of SNP base-pair positions. The input file can be selected interactively or manually specified by the user (see below).

Arguments:

`k`	integer, indicates the block size. Data are divided in blocks containing `k` SNPs each. If the number of SNPs is not a multiple of block size, the remaining SNPs are discarded (as TreeMix does).
`refline`	integer or an integer vectors, indicates one or more reference lines representing physical distances of interest, expressed in kilobase pairs (kb). Set `refline=NULL` or ignore it to suppress its use.
`int`	logical, specifies if choose the input file interactively or not. Default: `TRUE`.
`inputfile`	character string, specifies the name of the input file when `int=FALSE`. If no path is specified, the input file must be placed in the current R working directory. The argument is ignored when `int=TRUE`.
`colP`	character string, allows the user to specify the colour of plotted points. See `?points()` for details (default: `colP= 'green2'`).
`pchP`	integer, allows the user to specify the shape of plotted points. See `?points()` for details (default: `pchP= 19`).
`colL`	character string, allows the user to specify the colour(s) for reference line(s). See ?`par()` for details (default: `colL='red2'`; values are recycled as needed).
`typeL`	integer, allows the user to specify the line type(s) for the reference line(s). See `?par()` for details (default: `typeL= 2`; values are recycled as needed).

Details: this function plots the distance (kb) between the midpoints of consecutive SNP blocks found on the same chromosome vs. the number of observed pairwise distances. Because distances between blocks entirely placed on different chromosomes are not calculated, the number of observed pairwise

distances could be smaller than expected. When a block contains SNPs belonging to two different chromosomes, the distances between neighbouring blocks (i.e., between their midpoints) are calculated using only SNPs from the chromosome of interest.

Output: a scatterplot of the distance between the midpoints of consecutive block pairs vs. the number of observed pairwise distances is plotted in the active graphics window. If all drawn points are above the reference line (that should represent meaningful value in terms of LD decay, see warnings below), than the selected block size could represent a reasonable choice for the -*k* parameter in TreeMix.

Usage: `BlockDist(K = 50, reflines = c(200,400), colP = 'purple', colL = c('red','black'))`

These commands set the block size to 50 SNPs, draw two reference lines, the first one in red at 200 000 bp and the second in black at 400 000 bp, and set dot colour to purple.

WARNING: since the function uses the difference between the midpoints of consecutive blocks as a proxy for the distance between SNPs belonging to different blocks, it is advised to choose reference values (i.e., `refline` argument) that exceeds the known extent of LD decay in the organism(s) of interest (see TreeMix manual).

- **cfTrees**(input_stem, nt, p=1, uel=TRUE, m='PH85')

Context: usually, several hundreds of ML tree searches have been performed using TreeMix and trees need to be quickly screened for differences in log-likelihoods and topologies before to proceed with the analysis of migrations. Also useful to compare the main tree topologies (i.e., not including migration edges in comparison) obtained allowing migration events.

Assumptions: input files are standard treemix.llik and treemix.treeout.gz files (see TreeMix manual) and MUST be placed in the current R working directory. All file input file MUST follow the same general naming convention:

file stem + a progressive number (starting from 1) + file extension (.llik/.treeout.gz).

Examples:

- filename_1.llik, filename_2.llik ...... filename_300.llik.
- filename_1.treeout.gz, filename_2.treeout.gz ...... filename_300.treeout.gz.

where filename_ = file stem.

Arguments:

`input_stem`	character, it is the input file stem. It must be the same as -o flag used to designate the output stem in TreeMix analysis.
`nt`	integer indicating the number of 'treemix.llik'/'treemix.treeout.gz' files contained in the current R working directory.
`p`	a numeric value between 0.5 and 1 giving the proportion for a clade to be represented in the consensus tree. Set p=1 (default) to compute the strict-consensus tree, set p=0.5 to get the majority-rule consensus tree.
`uel`	logical specifying whether to consider the edge lengths in comparisons when removing duplicate trees. By default the edge lengths are compared here. This behaviour differs from the default in the `unique.multiPhylo()` function (see the package ‘ape‘ manual for details).
`m`	a character string giving the method to be used in computing topological distances between phylogenetic trees: either "PH85", or "score". Default: "PH85" (i.e., Robinson-Foulds distance). See `dist.topo()` function in the package ‘ape‘ for explanations on the available methods.

Details: the function compares all trees found in the current R working directory considering their likelihoods and topologies. First, trees are ordered based on their likelihoods, and the best known maximum likelihoods trees are identified. Then, duplicate trees are removed (see the `uel` argument

above) and the topological distances between the retained ML trees are computed using the method selected by the user (see `m` argument above). A consensus tree is also computed (see the `p` argument above for details). A summary of results is printed to the console and several files are saved in the current working directory (see "Output" below).

Output: the consensus tree obtained from non-redundant ML topologies is printed in the active graphics window and a summary of results is printed to the console output, including:

- the number of trees read as input;
- the IDs of the best-known ML trees;
- the number of duplicates in the best-known ML trees;
- the IDs of duplicates in ML trees;
- the number of unique ML trees;
- the IDs of unique ML tree(s);
- a matrix showing pairwise topological distances between unique ML trees.

In addition, several files are save in the current working directory:

- "TreeLLs.txt": log-likelihood scores shown in descending order (all trees);
- "AllBestTrees.newick": best-known ML trees, including duplicates (newick format);
- "UniqueTop.newick": best-known ML trees with unique topologies (newick format);
- "PairwDist": showing pairwise topological distances between unique ML trees;
- "Consensus.newick": the consensus tree obtained from the unique ML trees (newick format).

Usage: `cfTrees("MLtree_",nt = 200,p=1, m='PH85')`: the function takes 200 ML trees called "MLtree_1"..."MLtree_200", selects trees with the highest likelihood, discards duplicates, computes their strict consensus tree and the corresponding pairwise distance matrix using the "PH85" method.

- **GetPairsOfSets**(outputfile = 'PairsOfSets.txt', skipL = 1)

Context: multiple independent TreeMix runs have provided similar, but not identical results. Usually (though not necessarily for this function to work) the *-k* value used and the number of migration events fitted are the same across independent runs.

Assumption: all outstem.treeout.gz files obtained from different runs are placed in the current R working directory.

Arguments:

`outputfile`	character specifying the name of the output file. Default value: PairsOfSets.txt. `""` indicates output to the console.
`skipL`	integer, the number of lines of the input files (outstem.treeout.gz files) to skip before beginning to read data values. Default = 1; set it to 2 when using –`noss` option in TreeMix analysis.

Details: the TreeMix output file named outstem.treeout.gz (`-o outstem` in TreeMix) includes the fitted tree and the inferred migration edges. Each migration event is represented by using two subtrees: the subtree below the origin of the migration edge and the subtree below the destination of the migration edge (see TreeMix manual). This function extracts all pairs of ordered sets of species names associated with migration events from the outstem.treeout.gz files found in the current R working directory and then removes duplicates. The remaining pairs of sets represent unique combinations between the tip labels found in the subtrees below the origins of migration edges and the tip labels found in the subtrees below the destinations of migration edges. Sets in a pair can be either disjoint sets or a set and one of its proper subsets. Within each set of a pair, tips labels are ordered alphabetically. Branching pattern and branch lengths are discarded during the process.

Output: the output is a two columns text file named PairsOfSets.txt (default). Columns headers are "From" and "To" indicating the set of species names below the origin of migration edges and the set of species names below the destination of the migration edge, respectively.

Usage:

`GetPairsOfSets()` default options

`GetPairsOfSets(outputfile= "")` to print output to the console.

- **GetMigrSupp**(m_from = "all", m_to = "all", inputfile = 'PairsOfSets.txt', outputfile = 'MigrSupp.txt', skipL = 1)

Context: *n* bootstrap replicates have been generated by TreeMIx using *the same -k value* and fitting the *same number of migrations* each time.

Assumptions: all n bootstrap replicates named filename_X.treeout.gz (where X = 1...*n*) are placed in the current R working directory. If `m_from` and `m_to` arguments are set to "`all`" (default, see below) the output file produced by the `GetPairsOfSets()` function must be in the same directory.

Arguments:

`m_from, m_to`	characters or character vectors. Pair(s) of label sets representing migration(s) of interest. Sets MUST be specified following the same format used in the output file produced by the `GetPairsOfSets()` function (including semicolons!). Default value: "`all`".
`inputfile`	character string specifying the name of the file used as input when `m_from = "all"` and `m_to = "all"`. It must be in the same format of the of the `GetPairsOfSets()` output file. Default value:"PairsOfSets.txt".
`outputfile`	character string specifying the name of the output file. Default value: MigrSupp.txt. `""` indicates output to the console.
`skipL`	integer, the number of lines of the outstem.treeout.gz files to skip before beginning to read data values. Default = 1; set it to 2 when using `-noss` option in TreeMix analysis.

Details: The function computes the percentage of times each pair of label sets is present within the *n* bootstrap replicates, as specified by the arguments `m_from` and `m_to`. This value is taken as migration support (MS) for the corresponding migration edge identified by the specified pair of sets. If `m_from` and `m_to` arguments are set to `"all"` (default), all pairs of sets present in the output file of the `GetPairsOfSets()` function are considered. The function looks for and counts only pairs of sets that are identical to those specified by the `m_from` and `m_to` arguments simultaneously (no partial overlap is allowed). Branching pattern information is ignored as well as branch lengths. Moreover, labels are alphabetically ordered before comparisons. For example, subtrees (A, (B, C) and (B (A, C)) are considered equivalent and support the same set of labels (A, B, C).

Output: the output is a three columns text file (default). Columns are named "From", "To" and "MS" indicating the subset of species below the origin of migration edges, the subset of species below the destination of migration edges and the MS values computed for migration edges based on bootstrap replicates.

Usage:

`GetMigrSupp()` default options

`GetMigrSupp(inputfile = 'your_input_file_name.txt', outputfile = 'yuor_output_file_name.txt')` specify input and output names

`GetMigrSupp(m_from = "(A,B,C);", m_to = "(D,E);")` ask for the MS for the migration edge that starts above the subtree (A, B, C) (whatever the inner branching pattern is) and that ends above the subtree (D, E).

Examples: suppose you have obtained from the `GetPairsOfSets()` function the following pair of sets: (A, B, C, D) → (E, F). You can obtain the support for exactly this migration typing: `GetMigrSupp(m_from="(A,B,C,D);", m_to="(E,F);")`.
However, you may be interested in knowing whether similar, but not identical, migrations appear within bootstrap replicates. For example, depending on the bootstrap replicate, this migration may be found as: (A, B, C, D) →(F) or (A, B, C, D) → (E) or (B, C, D) → (E, F) ... and so on.
To get alternative supports simply type:

```
GetMigrSupp(m_from="(A,B,C,D);", m_to="(F);")
GetMigrSupp(m_from="(A,B,C,D);", m_to="(E);")
GetMigrSupp(m_from="(B,C,D);", m_to="(D,E);")
```
... and so on.

You can also specify multiple pairs of sets (i.e., migrations) at once using character vectors:

```
GetMigrSupp(m_from=c("(A,B,C,D);",  "(A,B,C,D);"  ,"(B,C,D);"),
         m_to=c("(F);" , "(E);" , "(D,E);"))
```

WARNING:
- Semicolons are mandatory!

Hint: use the `GetExtMigrSupp() function` to search for multiple pairs of sets that support the same migration edge at once while correcting for multiple matches.

```
• GetExtMigrSupp(skipL=1, min_n = 1, nmigr, m_from, m_to, fixed = "To", inputfile = 'PairsOfSets.txt', outputfile = 'ExtMigrSupp.txt')
```

Context: *n* bootstrap replicates have been generated by TreeMIx using the *same -k value* and fitting the *same number of migrations* each time.

Assumption: all *n* bootstrap replicates named filename_X.treeout.gz (with X = 1...*n*) are placed in the current R working directory. If `m_from` and `m_to` arguments are set to `"all"` (see below) the output file produced by the `GetPairsOfSets()` function must be in the same directory.

Arguments:

`skipL`	integer, the number of lines of the outstem.treeout.gz files to skip before beginning to read data values. Default = 1; set it to 2 when using `-noss` option in TreeMix analysis.
`m_from`, `m_to`	character or characte vectors. Pair(s) of label sets representing migration(s) of interest. Sets MUST be specified following the same format used in the output file produced by the `GetPairsOfSets()` function (including semicolons!!!). Set *both* `m_from` and `m_to` arguments to `"all"` to analyze ALL pairs of sets included in the output of the `GetPairsOfSets()` function or in a file with the same format (see below).
`min_n`	integer representing the minimum number of elements (species labels) to be included within the unfixed set(s) (see below). Default value: `1`
`fixed`	character string. Only two values are possible: `"From"` or `"To"`. This argument specifies which label set of each pair r must be kept fixed during the analysis (see Details below). `Fixed ="From"` fixes the origin of the migration (s) of interest; `Fixed = "To"` fixes the destination of the same migration (s). Default value: `"To"`.
`nmigr`	integer specifying the number of migration events fitted by TreeMix.
`inputfile`	character string specifying the name of the input file to be used when *both* `m_from` and `m_to` arguments are set to `"all"`. Otherwise the argument is ignored by the function. The input file must be in the same format of the `GetPairsOfSets()` output file. Default value: PairsOfSets.txt.
`outputfile`	character string specifying the name of the output file. Default name: ExtMigrSupp.txt. "" indicates output to the console.

Details: This function relaxes the definition of migration support introduced with the `GetMigrSupp()` function. As in the previous function, migrations of interest are specified as pairs of sets via the `m_from` and `m_to` arguments. For each pair of sets specified by the user, the `fixed` argument "fixes" the set of labels representing the origin of the migration edge (`fixed="From"`) or the set of labels corresponding to the destination of the migration edge (`fixed="To"`). For the "fixed set", the function does an exact match search in all bootstrap replicates, exactly as the function `GetMigrSupp()` does. The "unfixed set" is treated differently: in fact not only the specified set, but also all its proper subsets return a positive match (default). This behaviour can be changed through the `min_n` argument which sets the minimum number of elements of a subset for this to be considered valid. For example, when `min_n = 3` and `fixed= "To"`, only subsets of `m_from` with at least 3 elements (taxa labels) are taken into account. If `min_n = length(m_from)`, this function is equivalent to the `GetMigrSupp()` function.

The function is rather flexible because it can analyse a single pair of sets as well as multiple pairs of sets at once, provided that the `min_n` and `fixed` arguments are correctly specified as vectors containing the appropriate values for each pair of sets analysed. To achieve this, they MUST have the same *length* and follow the same *order* of the `m_from` and `m_to` arguments. Failure to do so could lead to misleading results! If `m_from` and `m_to` arguments are set to `"all"`, all pairs of sets found in the input file are analysed. Note that if different TreeMix runs have lead to different combinations of migrations, the number of set pairs present in the input file is > `nmigr` and the `m_from` and `m_to` arguments must be set accordingly.

As for MS index, the extended MS index is calculated as: (number of matches / number of bootstrap replicates)*100. In the latter however, the number of counts is corrected for multiple matches to avoid over counting.

Value: the output is a five columns text file (default). Columns are named "From", "To", “TotalCount”, ”CorrectedCount” and "ExtBS" indicating: the subset of species below the origin of the migration edges, the subset of species below the destination of migration edges, the total number of matches, the corrected number of matches and the MS_E computed based on bootstrap replicates. In addition, a summary of results is printed to the console in the form of a table in which migration edges are indicated as “migr#1”, “migr#2”, …. “migr# *n*”, following the same order specified by the user.

Usage:

```
GetExtMigrSupp(min_n = 2, nmigr =5, m_from="(A,B,C);", m_to="(D,E);"
fixed = "To", outputfile = 'yuor_output_file_name')
```

The function fixes the set specified by the `m_to` argument, counts all subsets of `m_from` with at least 2 elements as valid matches and changes the name of the output file. Five migration events are assumed.

`GetExtMigrSupp(min_n = c(3,1), m_from = c("(F,G,H,I);","(A,B,C);"), m_to = c("(L,M);","(D,E);"), fixed = c("To", "From", nmigr = 7)`

The function computes the extended migration support for the two migration edges described by the pairs of sets: (A, B, C) → (D, E) and (F, G, H, I) → (L, M). Different values for the `min_n and` the `fixed` arguments are used. All subset of (F, G, H, I) with at least 3 elements and all subsets and all subsets of (D, E) with at least 1 element are considered as valid matches to support the specified migration edges. Seven migrations events are assumed.

Examples: suppose you have obtained the following a pair of sets (A, B, C, D) → (E, F) using the `GetPairsOfSets()` function. You can obtain the support for exactly this migration typing: `GetExtMigrSupp(min_n=4, m_from="(A,B,C,D);", m_to="(E,F);", fixed = "To")` or equivalently typing `GetExtMigrSupp(min_n=2, m_from ="(A,B,C,D);", m_to="(E,F);", fixed = "From").`

However, you may be interested in considering similar, but not identical, migrations that may be present in your bootstrap replicates. For example, you may want to count not only the migration edge (A, B, C, D) → (E, F), but also the somewhat similar edges (A, B, C, D) → (E) and (A, B, C, D) → (F). You can do this simply typing:

`GetExtMigrSupp(min_n=1, m_from="(A,B,C,D);", m_to="(E,F);", fixed = "From", nmigr = 7)`. Thus, the extended support here is the sum of the support of three different edges. This is substantially different from the `GetMigrSupp()` function where the support for each migration edge is evaluated individually.

Likewise, typing:

`GetExtMigrSupp(min_n=3, m_from="(A,B,C,D);", m_to="(E,F);", fixed = "To", nmigr = 7)` all the following migration edges are theoretically included in the computation of the extended support: (A, B, C, D) → (E, F) ; (A, B, C) → (E, F) ; (A, C, D) →(E, F) ; (A, B, D) → (E, F) ; (B, C, D) → (E, F).However, since they represent the tips of different subtrees, they are not necessarily ALL found within your bootstrap replicates.

Because of the random nature of bootstrap replicates and because there may be a partial overlap between two or more pairs of sets, relaxing the matching criterion may results in multiple matches per replicate. Unless we are interested in a sort of "relative strength" of migrations, this is not be desirable. For this reason the MS_E is computed correcting for multiple matches to avoid over counting. To further inspect multiple matches the function called `MultiOccurrCheck()` can be used.

```
• MultiOccurrCheck(skipL = 1, nmigr, target, where = "To",
  extout = TRUE, outputfile = 'MultipleOccurences.txt')
```

Context: *n* bootstrap replicates have been generated by TreeMix using the *same -k value* and fitting the *same number of migrations* each time. Using the function `GetExtMigrSupp()` we have discovered that multiple matches are present and we are interested in investigating bootstrap replicates containing multiple occurrences.

Assumption: all *n* "bootstrap_replicate_X.treeout.gz" (with X = 1...*n*) files are placed in the current R working directory.

Arguments:

`skipL`	integer, the number of lines of the outstem.treeout.gz files to skip before beginning to read data values. Default = 1; set it to 2 when using `-noss` option in TreeMix analysis.
`Nmigr`	integer, indicating the number of migration events fitted during bootstrap analysis (it MUST be the same in all replicates)
`target`	character string specifying the "target set" to be checked for multiple occurrences, normally the "fixed" set in the `GetExtMigrSupp()function` (see below). It must be specified following the same format used in the output file produced by the `GetPairsOfSets()` function (including semicolons!).
`where`	character string. This argument specifies whether the "target set" must be searched within the label sets representing the origin of migrations or the label sets representing the destination of migrations. Only two values are possible: `"From"` or `"To"`.Default value: "To".
`extout`	logical indicating whether the output must be in the extended output format (TRUE; default option) or in the minimal output format (FALSE). The minimal output format prints on screen the names of replicates containing multiple occurrences of the specified "target set", together with the number of occurrences found within in each file. The extended output format prints on screen the same minimal output. In addition, all pairs of label sets including the "target set" are extracted from files, grouped by replicate and saved to the output file (or printed to the console, see `outputfile`).
`Outputfile`	character string, specifying the name of the output file. Default value: MultipleOccurences.txt. `""` indicates output to the console.

Details: this function for inspects all bootstrap replicates found in the current R working directory looking for multiple occurrences of the specified “target set". The "target set" is specified via the `target` argument and the search can be done either within the label sets representing the origin of migrations or the label sets representing the destination of migrations, depending on the value assigned to the argument `where`. Thought not necessary to calculate migration support, this function allow the user to investigate the reasons underlying multiple matches.

Value: the output is a two columns matrix printed on screen showing bootstrap replicates with multiple occurrences of the specified "target set". The number of occurrences found within in each file is also shown. If `extout=TRUE` (default) a file containing all pairs of label sets including the "target set" of interest is saved in the current R working directory.

WARNING: the `GetExtMigrSupp()` function returns a positive match only for the specified set and all its proper subsets> `min_n`, whereas the `MultiOccurrCheck()` function counts and extracts ALL possible multiple occurrences matching the specified constraints (i.e., including those due to different sets!). Thus, it is necessary that the extended output of the `MultiOccurrCheck()` function is carefully checked to understand the reasons underlying multiple matches.

Usage:

```
MultiOccurrCheck(nmigr = 4,target ="(A,B,C);")
```

The function looks for "(A, B, C);" within the label sets representing the destination of migrations. Four migration events were fitted during bootstrap analysis. The extended output format is required and all pairs of label sets including the "target set" of interest are saved to a file named MultipleOccurences.txt.

```
MultiOccurrCheck(nmigr = 8, target = "(A,B,C,D,E);", where = "From",
                 extout = FALSE)
```

The function looks for "(A, B, C, D, E);" within the label sets representing the origin of migrations. Eight migration events were fitted during bootstrap analysis. The minimal output format is printed on screen.

Example: suppose you have run 100 boostrap replicates and you are interested in knowing the extended bootstrap support of (A, B, C, D) → (E, F) edge.

Calling: `GetExtMigrSupp(min_n =1, nmigr = 4, m_from="(A,B,C,D);", m_to="(E,F);", fixed = "From")` you have discovered that multiple matches are present within bootstrap replicates.

Typing: `MultiOccurrCheck(nmigr = 4, target = "(A,B,C,D);",where = "From")` you can investigate the reason of multiple matches (here 4 migration events are assumed).

For example, you might find that (A, B, C, D) → (E) and (A,B,C, D)--->(F) edges are both present within some bootstrap replicates (i.e., double counts) while other replicates contain all the three possible edges: (A, B, C, D) → (E, F), (A, B, C, D) → (E) and (A, B, C, D) → (F) (i.e., triple counts).

6. References

Inference of Population Splits and Mixtures from Genome-Wide Allele Frequency Data Pickrell JK, Pritchard JK (2012) Inference of Population Splits and Mixtures from Genome-Wide Allele Frequency Data. PLOS Genetics 8(11): e1002967. https://doi.org/10.1371/journal.pgen.1002967

Purcell S, Neale B, Todd-Brown K, Thomas L, Ferreira MAR, Bender D. et al., (2007) PLINK: a toolset for whole-genome association and population-based linkage analysis. American Journal of Human Genetics 81(3): 559–75
