# Supplementary material for: Untangling the Evolution of American Wild Grapes: Admixed Species and How to Find Them
Source: Front Plant Sci. 2020 Feb 7;10:1814. doi: 10.3389/fpls.2019.01814 (PMC7025467; doi:10.3389/fpls.2019.01814)
Supplement: Supplementary file 1 [file DataSheet_1.zip › Supplementary material/Supplementary file S2/readme.docx]

The file named "RAxML_BestTree" contains the best-scoring ML-tree out of 100 independent ML searches performed in RAxML-HPC2 v. 8.2.10 under GTRCAT model of substitution, with -c set to 25, -i determined automatically by RAxML and the --asc-corr option set to lewis.

Random maximum parsimony starting trees were used to initialize ML tree searches.

The file has been modified using FigTree to highlight the names of the discarded accessions in red. Use FigTree software (http://tree.bio.ed.ac.uk/software/figtree/) to visualise.
