# Supplementary material for: Untangling the Evolution of American Wild Grapes: Admixed Species and How to Find Them
Source: Front Plant Sci. 2020 Feb 7;10:1814. doi: 10.3389/fpls.2019.01814 (PMC7025467; doi:10.3389/fpls.2019.01814)
Supplement: Supplementary file 1 [file DataSheet_1.zip › Supplementary material/Supplementary Tables and Figures.pdf]

Supplementary Material

- **Supplementary Tables**
  - **Supplementary Table S1** ……………………………………….…….. **p. 3**
  - **Supplementary Table S2** ………………..…………………….…… **p. 4**
  - **Supplementary Table S3** ……………………………………….….. **p. 7**
  - **Supplementary Table S4 A-H** …...…………………………….… **p. 8**

- **Supplementary Figures**
  - **Supplementary Figure S1** ……………………………………………… **p. 17**
  - **Supplementary Figure S2** …..……………………………………….. **p. 20**
  - **Supplementary Figure S3** …..……………………………………….. **p. 21**

- **References** ……………………………………..………………..….. **p. 22**

Supplementary Tables

Table S1. Accessions of cultivated grapevine included in this work. Accession IDs refer to Germplasm Resources Information Network (GRIN) Plant Taxonomy (USDA 2019). Cultivar names based on Myles et al. (2011).

	SPECIES	ID	CULTIVAR
1.	*V. vinifera* subsp. *vinifera*	DVIT_1024	Agoumastos
2.	*V. vinifera* subsp. *vinifera*	DVIT_645	Assirtico
3.	*V. vinifera* subsp. *vinifera*	DVIT_2087	Aswad
4.	*V. vinifera* subsp. *vinifera*	DVIT_657	Berzamino
5.	*V. vinifera* subsp. *vinifera*	DVIT_1081	Bhokri
6.	*V. vinifera* subsp. *vinifera*	DVIT_671	Bonarda
7.	*V. vinifera* subsp. *vinifera*	DVIT_677	Cabernet Sauvignon
8.	*V. vinifera* subsp. *vinifera*	DVIT_950	Chardonay
9.	*V. vinifera* subsp. *vinifera*	DVIT_707	Dolcetto
10.	*V. vinifera* subsp. *vinifera*	DVIT_577	Italia (IP 65)
11.	*V. vinifera* subsp. *vinifera*	DVIT_579	Julius Caesar
12.	*V. vinifera* subsp. *vinifera*	DVIT_774	Katta Kurgan
13.	*V. vinifera* subsp. *vinifera*	DVIT_786	Kristalli
14.	*V. vinifera* subsp. *vinifera*	DVIT_2117	Malvasia Bianca
15.	*V. vinifera* subsp. *vinifera*	DVIT_812	Malvasia Roxa
16.	*V. vinifera* subsp. *vinifera*	DVIT_818	Marzemino
17.	*V. vinifera* subsp. *vinifera*	DVIT_2682	Merlot
18.	*V. vinifera* subsp. *vinifera*	DVIT_2337	Mueller Thurgau
19.	*V. vinifera* subsp. *vinifera*	DVIT_853	Mueller-Thurgau
20.	*V. vinifera* subsp. *vinifera*	DVIT_468	Muscat Hamburg
21.	*V. vinifera* subsp. *vinifera*	DVIT_841	Muscat of Alexandria
22.	*V. vinifera* subsp. *vinifera*	DVIT_2643	Muscat Oliver
23.	*V. vinifera* subsp. *vinifera*	DVIT_867	Nebbiolo
24.	*V. vinifera* subsp. *vinifera*	DVIT_872	Negro Amaro
25.	*V. vinifera* subsp. *vinifera*	DVIT_1065	Pinot Blanc
26.	*V. vinifera* subsp. *vinifera*	DVIT_907	Pinot Gris
27.	*V. vinifera* subsp. *vinifera*	DVIT_945	Sangiovese
28.	*V. vinifera* subsp. *vinifera*	DVIT_2653	Sereksiya Rosavi
29.	*V. vinifera* subsp. *vinifera*	DVIT_526	Sultana
30.	*V. vinifera* subsp. *vinifera*	DVIT_530	Sultanina Rose
31.	*V. vinifera* subsp. *vinifera*	DVIT_1053	Syrah
32.	*V. vinifera* subsp. *vinifera*	DVIT_977	Teroldego
33.	*V. vinifera* subsp. *vinifera*	DVIT_2157	Tokay
34.	*V. vinifera* subsp. *vinifera*	DVIT_991	Traminer
35.	*V. vinifera* subsp. *vinifera*	DVIT_2086	Vranac
36.	*V. vinifera* subsp. *vinifera*	DVIT_1340	Zinfandel

Table S2. List of American accessions excluded from the analysis, with reasons for exclusion. Accession IDs refer to GRIN Plant Taxonomy (USDA 2019).

	SPECIES	ID	REASON FOR EXCLUSION
1.	*V. aestivalis*	DVIT 119	American Hybrid [1]/ Hybrid with *V. vinifera* ancestry [3]
2.	*V. aestivalis*	DVIT 1446	Low genotyping [5]
3.	*V. aestivalis*	DVIT 1855	Hybrid with *V. vinifera* ancestry [4]
4.	*V. aestivalis*	DVIT 1916	Hybrid with *V. vinifera* ancestry [4]
5.	*V. aestivalis* var. *aestivalis*	DVIT 1704	Hybrid with *V. vinifera* ancestry [3,4]
6.	*V. aestivalis* var. *aestivalis*	DVIT 1705	Hybrid with *V. vinifera* ancestry [3,4]
7.	*V. aestivalis* var. *aestivalis*	DVIT 1711	Hybrid with *V. vinifera* ancestry [3]
8.	*V. aestivalis* var. *aestivalis*	DVIT 1717	Hybrid with *V. vinifera* ancestry [3,4]
9.	*V. aestivalis* var. *aestivalis*	DVIT 1718	Hybrid with *V. vinifera* ancestry [3,4]
10.	*V. aestivalis* var. *aestivalis*	DVIT 2099	Hybrid with *V. vinifera* ancestry [3,4]
11.	*V. aestivalis* var. *aestivalis*	DVIT 2720	Hybrid with *V. vinifera* ancestry [3,4]
12.	*V. aestivalis* var. *bicolor*	PI 483137.02	Possible mislabeling/misidentification [4]
13.	*V. aestivalis* var. *lincecumii*	DVIT 1394	American Hybrid [1]
14.	*V. aestivalis* var. *lincecumii*	DVIT 1395	American Hybrid [1]
15.	*V. californica*	DVIT 1361	Hybrid with *V. vinifera* ancestry [4]
16.	*V. californica*	DVIT 1836	Hybrid with *V. vinifera* ancestry [4]
17.	*V. champinii*	DVIT 1386	Misidentification[7]
18.	*V. champinii*	DVIT 1388	Misidentification[7]
19.	*V. cinerea*	DVIT 1362	American Hybrid [1]
20.	*V. cinerea*	DVIT 1363	American Hybrid [1]
21.	*V. cinerea*	DVIT 1364	Hybrid with *V. vinifera* ancestry [1]
22.	*V. cinerea*	DVIT 1365	American Hybrid [1]
23.	*V. cinerea*	DVIT 1875	Hybrid with *V. vinifera* ancestry [3,4]
24.	*V. cinerea* var. *helleri*	DVIT 2224.8	American Hybrid [1]
25.	*V. cinerea* var. *helleri*	PI 588466.02	Donated from France[1]
26.	*V. girdiana*	DVIT 1387	American Hybrid [1]
27.	*V. labrusca*	DVIT 102	American Hybrid [1]/ Hybrid with *V. vinifera* ancestry [3]
28.	*V. labrusca*	DVIT 1129	Hybrid with *V. vinifera* ancestry [1,2,3]
29.	*V. labrusca*	DVIT 124	Hybrid with *V. vinifera* ancestry [3,4]
30.	*V. labrusca*	DVIT 130	American Hybrid [1]
31.	*V. labrusca*	DVIT 1391	American Hybrid [1]
32.	*V. labrusca*	DVIT 1392	American Hybrid [1]
33.	*V. labrusca*	DVIT 1393	American Hybrid [1]
34.	*V. labrusca*	DVIT 162	American Hybrid [1]/ Hybrid with *V. vinifera* ancestry [3]
35.	*V. labrusca*	DVIT 168	American Hybrid [1]/ Hybrid with *V. vinifera* ancestry [3]
36.	*V. labrusca*	DVIT 31	American Hybrid [1]/ Hybrid with *V. vinifera* ancestry [3]
37.	*V. labrusca*	DVIT 40	American Hybrid [1]/ Hybrid with *V. vinifera* ancestry [3]
38.	*V. labrusca*	DVIT 66	American Hybrid [1]/ Hybrid with *V. vinifera* ancestry [3]
39.	*V. labrusca*	DVIT 69	American Hybrid [1]/ Hybrid with *V. vinifera* ancestry [3]
40.	*V. labrusca*	DVIT 71	American Hybrid [1]/ Hybrid with *V. vinifera* ancestry [3]

41.	*V. labrusca*	DVIT 81	American Hybrid [1]/ Hybrid with *V. vinifera* ancestry [3]
42.	*V. labrusca*	DVIT 87	Hybrid with *V. vinifera* ancestry [3,4]
43.	*V. labrusca*	GVIT 1587.02	Tetraploid, Hybrid with V. vinifera ancestry [1,3]
44.	*V. labrusca*	PI 588128.02	Hybrid with *V. vinifera* ancestry [1,2,3]
45.	*V. labrusca*	PI 588169.02	Hybrid with *V. vinifera* ancestry [3,4]
46.	*V. labrusca*	PI 588277.02	Suspected American hybrid / Hybrid with *V. vinifera* ancestry [6]
47.	*V. labrusca*	PI 588307.02	Suspected American hybrid / Hybrid with *V. vinifera* ancestry [6]
48.	*V. labrusca*	PI 588658.06	Cultivar of interspecific ancestry [1,2]
49.	*V. labrusca*	PI 588675.02	Hybrid with *V. vinifera* ancestry [3]
50.	*V. labrusca*	PI 594349.02	Hybrid with *V. vinifera* ancestry [1,2,3]
51.	*V. labrusca*	PI 597172.02	Hybrid with *V. vinifera* ancestry [3]
52.	*V. labrusca*	PI 597203.02	Hybrid with *V. vinifera* ancestry [1,2,3]
53.	*V. labrusca*	PI 597228.02	Hybrid with *V. vinifera* ancestry [3]
54.	*V. mustangensis*	DVIT 1577	Cultivar of interspecific ancestry [1,2]
55.	*V. riparia*	DVIT 128	Hybrid with *V. vinifera* ancestry [3,4]
56.	*V. riparia*	DVIT 1411	Hybrid with *V. vinifera* ancestry [3,4]
57.	*V. riparia*	DVIT 1425	Donated from France[1]
58.	*V. riparia*	DVIT 1432	Possible mislabeling/misidentification[1,2]
59.	*V. riparia*	DVIT 1848	Hybrid with *V. vinifera* ancestry [3,4]
60.	*V. riparia*	DVIT 2092	Hybrid with *V. vinifera* ancestry [4]
61.	*V. riparia*	PI 313922.02	Donated from Russia Federation[1] / *V. vinifera*[2]
62.	*V. riparia*	PI 588190.02	Cultivar of interspecific ancestry [1,2]
63.	*V. riparia*	PI 588436.02	Hybrid with *V. vinifera* ancestry [3,4]
64.	*V. rotundifolia*	DVIT 1145	Unknown Euvitis cultivar[1]
65.	*V. rotundifolia*	DVIT 1723	Cultivar of interspecific ancestry [1,2]
66.	*V. rotundifolia*	DVIT 1726	Cultivar of interspecific ancestry [2]
67.	*V. rotundifolia*	DVIT 1747	Low genotyping [5]
68.	*V. rotundifolia*	DVIT 1753	Cultivar of interspecific ancestry [1,2]
69.	*V. rotundifolia*	DVIT 1760	Cultivar of interspecific ancestry [1,2]
70.	*V. rotundifolia*	DVIT 1761	Cultivar of interspecific ancestry [1,2]
71.	*V. rotundifolia*	DVIT 1792	Low genotyping [5]
72.	*V. rotundifolia*	DVIT 2191	Low genotyping [5]
73.	*V. rotundifolia*	DVIT 2439	Low genotyping [5]
74.	*V. rotundifolia*	DVIT 2628	Hybrid with *V. vinifera* ancestry [3,4]
75.	*V. rotundifolia*	DVIT 2974	Low genotyping [5]
76.	*V. rotundifolia*	NA	Unknown accession
77.	*V. rotundifolia* var. *munsoniana*	DVIT 1708	Possible mislabeling/misidentification [4]
78.	*V. rupestris*	DVIT 1599	Tetraploid [1]
79.	*V. rupestris*	PI 237621.02	Hybrid with *V. vinifera* ancestry [3,4]
80.	*V. rupestris*	PI 588454.g2	Donated from Russia Federation[1]
81.	*V. shuttleworthii*	DVIT 1719	Hybrid with *V. vinifera* ancestry [3,4]
82.	*V. tiliifolia*	DVIT 1716	Hybrid with *V. vinifera* ancestry [3,4]
83.	*V. treleasei*	DVIT 1149	American Hybrid [1]
84.	*V. vulpina*	DVIT 1289	Hybrid with *V. vinifera* ancestry [4]
85.	*V. vulpina*	DVIT 1368	American Hybrid [1]
86.	*V. vulpina*	DVIT 1381	American Hybrid [1]
87.	*V. vulpina*	DVIT 1382	American Hybrid [1]
88.	*V. vulpina*	DVIT 1637	Possible American Hybrid [1]
89.	*V. vulpina*	PI 483186.02	Hybrid with *V. vinifera* ancestry [4]

90.	*V. vulpina*	PI 588185.02	Hybrid with *V. vinifera* ancestry [3,4]

1= based on information determined from the GRIN Plant Taxonomy (http://www.ars-grin.gov/; accessed February 9, 2017).
2= based on information determined from the *Vitis* International Variety Catalogue (http://www.vivc.de/index.php; Maul et al. 2017; accessed February 9, 2017).
3= based on Sawler et al. (2013).
4 = based on our preliminary phylogenetic reconstruction (RAxML).
5 = based on dataset filtering (PLINK: MIND > 0.2).
6 = based on our preliminary phylogenetic reconstruction (RAxML) and linkage disequilibrium analysis.
7 = Cousins, P. (1999).

Table S3. Results of forty independent runs performed by TreeMix fitting nine admixture events. Two different ML trees were used to initialise the analyses. For each run the random seed (Seed#) used to initialise the analysis, the final log-likelihood [ln(likelihood)] and the variance explained by the model (f) are shown in the table.

Run#	Starting tree#	Seed#	ln(likelihood)	f
1	1	1572041337	2691.54	0.985782
2	1	1572042958	2691.54	0.985782
3	1	1572044575	2691.54	0.985782
4	1	1572046194	2691.54	0.985782
5	1	1572047760	2691.54	0.985782
6	1	1572049293	2691.54	0.985782
7	1	1572050836	2691.54	0.985782
8	1	1572052383	2691.54	0.985782
9	1	1572053994	2691.54	0.985782
10	1	1572055613	2691.54	0.985782
11	1	1572057187	2691.54	0.985782
12	1	1572058751	2691.54	0.985782
13	1	1572060274	2691.54	0.985782
14	1	1572061902	2691.54	0.985782
15	1	1572063517	2691.54	0.985782
16	1	1572065095	2691.54	0.985782
17	1	1572066675	2691.54	0.985782
18	1	1572068268	2691.54	0.985782
19	1	1572069887	2691.54	0.985782
20	1	1572071495	2691.54	0.985782
21	2	1572041427	2691.54	0.985782
22	2	1572043010	2691.54	0.985782
23	2	1572044670	2691.54	0.985782
24	2	1572046253	2691.54	0.985782
25	2	1572047848	2691.54	0.985782
26	2	1572049529	2691.54	0.985782
27	2	1572051107	2691.54	0.985782
28	2	1572052708	2691.54	0.985782
29	2	1572054310	2691.54	0.985782
30	2	1572055894	2691.54	0.985782
31	2	1572057458	2691.54	0.985782
32	2	1572059110	2691.54	0.985782
33	2	1572060763	2691.54	0.985782
34	2	1572062443	2691.54	0.985782
35	2	1572064024	2691.54	0.985782
36	2	1572065680	2691.54	0.985782
37	2	1572067333	2691.54	0.985782
38	2	1572068900	2691.54	0.985782
39	2	1572070479	2691.54	0.985782
40	2	1572072038	2691.54	0.985782

Table S4A-S4H. Details on migrations inferred by TreeMix during forty independent runs performed fitting nine admixture events. Two different ML trees were used to initialise the analyses. In the following tables capital letters A-H refer to migration edges A-H discussed in the main text and summarized in Table 1. For each independent run the random seed used to initialise the analysis, the weight on the edge (W), the jackknife estimate of the weight (W_j), the jackknife estimate of the standard error (SE_j) and the p-value computed by TreeMix are shown in the tables.

Table S4 A

Migration edge A					
**Run#**	**Starting tree#**	**W**	**W_j**	**SE_j**	**P-value**
1	1	0.491543	0.491628	0.0199241	<2.22507e-308
2	1	0.49157	0.491613	0.019824	<2.22507e-308
3	1	0.491557	0.491627	0.0197418	<2.22507e-308
4	1	0.491534	0.491632	0.020045	<2.22507e-308
5	1	0.491557	0.491627	0.0197418	<2.22507e-308
6	1	0.491554	0.491632	0.0198968	<2.22507e-308
7	1	0.491557	0.491627	0.0197418	<2.22507e-308
8	1	0.49157	0.491613	0.019824	<2.22507e-308
9	1	0.491551	0.491631	0.0199048	<2.22507e-308
10	1	0.491561	0.491628	0.0197321	<2.22507e-308
11	1	0.491543	0.491628	0.0199228	<2.22507e-308
12	1	0.491549	0.49163	0.0199093	<2.22507e-308
13	1	0.491561	0.491628	0.0197321	<2.22507e-308
14	1	0.49157	0.491613	0.019824	<2.22507e-308
15	1	0.491543	0.491628	0.0199239	<2.22507e-308
16	1	0.491554	0.491632	0.0198968	<2.22507e-308
17	1	0.491517	0.491643	0.0201246	<2.22507e-308
18	1	0.491551	0.491631	0.0199048	<2.22507e-308
19	1	0.491544	0.491628	0.0199202	<2.22507e-308
20	1	0.491555	0.491632	0.0198952	<2.22507e-308
21	2	0.491559	0.491633	0.0198857	<2.22507e-308
22	2	0.49154	0.491634	0.0200302	<2.22507e-308
23	2	0.491557	0.491632	0.0198912	<2.22507e-308
24	2	0.491547	0.491629	0.0199131	<2.22507e-308
25	2	0.491514	0.491643	0.0201319	<2.22507e-308
26	2	0.491551	0.491631	0.019904	<2.22507e-308
27	2	0.491578	0.491616	0.0198059	<2.22507e-308
28	2	0.491547	0.491629	0.0199151	<2.22507e-308
29	2	0.491548	0.49163	0.019912	<2.22507e-308
30	2	0.491512	0.491642	0.0201377	<2.22507e-308
31	2	0.491547	0.491629	0.0199131	<2.22507e-308
32	2	0.491543	0.491628	0.0199227	<2.22507e-308
33	2	0.491516	0.491643	0.0201278	<2.22507e-308
34	2	0.491578	0.491616	0.0198059	<2.22507e-308
35	2	0.49154	0.491627	0.0199298	<2.22507e-308
36	2	0.491559	0.491633	0.0198857	<2.22507e-308
37	2	0.491516	0.491643	0.0201278	<2.22507e-308
38	2	0.491548	0.49163	0.019912	<2.22507e-308
39	2	0.491516	0.491643	0.0201278	<2.22507e-308
40	2	0.491537	0.491633	0.0200379	<2.22507e-308

Table S4 B

Migration edge B					
Run#	Starting tree#	W	W_j	SE_j	P-value
1	1	0.443739	0.443911	0.0245721	<2.22507e-308
2	1	0.443736	0.4439	0.0246146	<2.22507e-308
3	1	0.443759	0.443922	0.0246614	<2.22507e-308
4	1	0.443763	0.44392	0.0246419	<2.22507e-308
5	1	0.443759	0.443922	0.0246614	<2.22507e-308
6	1	0.443755	0.443921	0.0246581	<2.22507e-308
7	1	0.443759	0.443922	0.0246614	<2.22507e-308
8	1	0.443736	0.4439	0.0246146	<2.22507e-308
9	1	0.443735	0.443909	0.0245683	<2.22507e-308
10	1	0.443735	0.443909	0.0245683	<2.22507e-308
11	1	0.443739	0.44391	0.0245716	<2.22507e-308
12	1	0.443759	0.443922	0.0246614	<2.22507e-308
13	1	0.443735	0.443909	0.0245683	<2.22507e-308
14	1	0.443736	0.4439	0.0246146	<2.22507e-308
15	1	0.443738	0.4439	0.0246167	<2.22507e-308
16	1	0.443755	0.443921	0.0246581	<2.22507e-308
17	1	0.443761	0.44392	0.0246402	<2.22507e-308
18	1	0.443735	0.443909	0.0245683	<2.22507e-308
19	1	0.443766	0.443921	0.024648	<2.22507e-308
20	1	0.443755	0.443921	0.0246581	<2.22507e-308
21	2	0.443752	0.44391	0.0247016	<2.22507e-308
22	2	0.443729	0.443907	0.0245617	<2.22507e-308
23	2	0.443735	0.4439	0.0246143	<2.22507e-308
24	2	0.44377	0.443922	0.0246516	<2.22507e-308
25	2	0.443752	0.44392	0.0246549	<2.22507e-308
26	2	0.443738	0.4439	0.0246165	<2.22507e-308
27	2	0.443735	0.4439	0.0246143	<2.22507e-308
28	2	0.443733	0.443909	0.0245659	<2.22507e-308
29	2	0.443761	0.44392	0.0246407	<2.22507e-308
30	2	0.443739	0.443912	0.0246215	<2.22507e-308
31	2	0.44377	0.443922	0.0246516	<2.22507e-308
32	2	0.443733	0.443909	0.0245659	<2.22507e-308
33	2	0.443739	0.443911	0.0245721	<2.22507e-308
34	2	0.443735	0.4439	0.0246143	<2.22507e-308
35	2	0.443772	0.443923	0.0246528	<2.22507e-308
36	2	0.443752	0.44391	0.0247016	<2.22507e-308
37	2	0.443739	0.443911	0.0245721	<2.22507e-308
38	2	0.443761	0.44392	0.0246407	<2.22507e-308
39	2	0.443739	0.443911	0.0245721	<2.22507e-308
40	2	0.443724	0.443908	0.0246096	<2.22507e-308

Table S4 C

Migration edge C					
**Run#**	**Starting tree#**	**W**	**W_j**	**SE_j**	**P-value**
1	1	0.153819	0.153812	0.0636858	0.00786412
2	1	0.153811	0.153808	0.063685	0.00786455
3	1	0.15384	0.153805	0.0636546	0.00784066
4	1	0.153831	0.153801	0.0636511	0.00783919
5	1	0.15384	0.153805	0.0636546	0.00784066
6	1	0.153834	0.15381	0.063648	0.00783374
7	1	0.15384	0.153805	0.0636546	0.00784066
8	1	0.153811	0.153808	0.063685	0.00786455
9	1	0.153857	0.153806	0.063556	0.00776006
10	1	0.153836	0.153804	0.0636534	0.00784018
11	1	0.153857	0.15381	0.0635559	0.00775874
12	1	0.15384	0.153805	0.0636546	0.00784066
13	1	0.153836	0.153804	0.0636534	0.00784018
14	1	0.153811	0.153808	0.0636818	0.00786213
15	1	0.153729	0.153816	0.0635315	0.00773692
16	1	0.153834	0.15381	0.063648	0.00783374
17	1	0.153741	0.153833	0.0634615	0.00767458
18	1	0.153857	0.153806	0.063556	0.00776006
19	1	0.153853	0.153816	0.0636597	0.00784134
20	1	0.153834	0.153805	0.0636309	0.00782153
21	2	0.153741	0.153817	0.063564	0.0077629
22	2	0.153834	0.153805	0.0636312	0.00782165
23	2	0.153857	0.153806	0.063556	0.00776006
24	2	0.153827	0.153804	0.0636501	0.00783742
25	2	0.153741	0.153823	0.0635895	0.00778175
26	2	0.153741	0.153813	0.0635141	0.00772385
27	2	0.153857	0.153806	0.063556	0.00776006
28	2	0.153853	0.153809	0.0635691	0.00776969
29	2	0.153867	0.153815	0.0635601	0.0077604
30	2	0.153857	0.153806	0.063556	0.00776006
31	2	0.153827	0.153804	0.0636501	0.00783742
32	2	0.153853	0.153808	0.063553	0.00775693
33	2	0.153819	0.153805	0.0636783	0.0078603
34	2	0.153857	0.153806	0.063556	0.00776006
35	2	0.153816	0.153819	0.0636815	0.00785803
36	2	0.153741	0.153817	0.063564	0.0077629
37	2	0.153819	0.153809	0.0636817	0.00786152
38	2	0.153867	0.153815	0.0635601	0.0077604
39	2	0.153819	0.153809	0.0636817	0.00786152
40	2	0.1538	0.153814	0.0636765	0.00785573

Table S4 D

Migration edge D					
**Run#**	**Starting tree#**	**W**	**W_j**	**SE_j**	**P-value**
1	1	0.431154	0.431088	0.0246273	<2.22507e-308
2	1	0.431164	0.431087	0.0246056	<2.22507e-308
3	1	0.431111	0.431092	0.0246711	<2.22507e-308
4	1	0.431082	0.431106	0.0246474	<2.22507e-308
5	1	0.431065	0.431111	0.0246166	<2.22507e-308
6	1	0.431111	0.431092	0.0246711	<2.22507e-308
7	1	0.431111	0.431092	0.0246711	<2.22507e-308
8	1	0.431164	0.431087	0.0246056	<2.22507e-308
9	1	0.431154	0.431088	0.0246273	<2.22507e-308
10	1	0.431082	0.431106	0.0246474	<2.22507e-308
11	1	0.431164	0.431087	0.0246056	<2.22507e-308
12	1	0.431029	0.431125	0.0247111	<2.22507e-308
13	1	0.431082	0.431106	0.0246474	<2.22507e-308
14	1	0.431154	0.431088	0.0246273	<2.22507e-308
15	1	0.431029	0.431125	0.0247111	<2.22507e-308
16	1	0.431111	0.431092	0.0246711	<2.22507e-308
17	1	0.431111	0.431092	0.0246711	<2.22507e-308
18	1	0.431154	0.431088	0.0246273	<2.22507e-308
19	1	0.431061	0.431103	0.0246341	<2.22507e-308
20	1	0.431029	0.431125	0.0247111	<2.22507e-308
21	2	0.43106	0.431112	0.0246417	<2.22507e-308
22	2	0.431068	0.431107	0.0246018	<2.22507e-308
23	2	0.431164	0.431087	0.0246056	<2.22507e-308
24	2	0.431065	0.431106	0.0246017	<2.22507e-308
25	2	0.431111	0.431092	0.0246711	<2.22507e-308
26	2	0.431111	0.431092	0.0246711	<2.22507e-308
27	2	0.431061	0.431106	0.0246689	<2.22507e-308
28	2	0.431164	0.431087	0.0246056	<2.22507e-308
29	2	0.431061	0.431106	0.0246689	<2.22507e-308
30	2	0.431154	0.431094	0.024898	<2.22507e-308
31	2	0.431065	0.431106	0.0246017	<2.22507e-308
32	2	0.431154	0.431088	0.0246273	<2.22507e-308
33	2	0.431154	0.431094	0.024898	<2.22507e-308
34	2	0.431061	0.431106	0.0246689	<2.22507e-308
35	2	0.431164	0.431087	0.0246056	<2.22507e-308
36	2	0.43106	0.431112	0.0246417	<2.22507e-308
37	2	0.431164	0.431087	0.0246056	<2.22507e-308
38	2	0.431168	0.431084	0.0245847	<2.22507e-308
39	2	0.431164	0.431087	0.0246056	<2.22507e-308
40	2	0.431068	0.431112	0.0246167	<2.22507e-308

Table S4 E

Migration edge E					
**Run#**	**Starting tree#**	**W**	**W_j**	**SE_j**	**P-value**
1	1	0.346687	0.346728	0.0547895	1.23878e-10
2	1	0.346666	0.346707	0.0547267	1.18482e-10
3	1	0.346649	0.346697	0.0547397	1.19778e-10
4	1	0.346666	0.346747	0.0548681	1.31084e-10
5	1	0.346666	0.346707	0.0547267	1.18482e-10
6	1	0.346649	0.346697	0.0547397	1.19778e-10
7	1	0.346649	0.346697	0.0547397	1.19778e-10
8	1	0.346666	0.346707	0.0547267	1.18482e-10
9	1	0.346687	0.346728	0.0547895	1.23878e-10
10	1	0.346666	0.346707	0.0547267	1.18482e-10
11	1	0.346666	0.346707	0.0547267	1.18482e-10
12	1	0.346628	0.346733	0.0548888	1.33341e-10
13	1	0.346687	0.346713	0.0546985	1.15923e-10
14	1	0.346687	0.346728	0.0547895	1.23878e-10
15	1	0.346628	0.346733	0.0548888	1.33341e-10
16	1	0.346649	0.346697	0.0547397	1.19778e-10
17	1	0.346649	0.346697	0.0547397	1.19778e-10
18	1	0.346687	0.346728	0.0547895	1.23878e-10
19	1	0.346666	0.346707	0.0547267	1.18482e-10
20	1	0.346628	0.346733	0.0548888	1.33341e-10
21	2	0.346628	0.346733	0.0548888	1.33341e-10
22	2	0.346628	0.346733	0.0548888	1.33341e-10
23	2	0.346666	0.346707	0.0547267	1.18482e-10
24	2	0.346666	0.346707	0.0547267	1.18482e-10
25	2	0.346649	0.346697	0.0547397	1.19778e-10
26	2	0.346649	0.346697	0.0547397	1.19778e-10
27	2	0.346666	0.346707	0.0547267	1.18482e-10
28	2	0.346666	0.346707	0.0547267	1.18482e-10
29	2	0.346666	0.346707	0.0547267	1.18482e-10
30	2	0.346687	0.346728	0.0547895	1.23878e-10
31	2	0.346666	0.346707	0.0547267	1.18482e-10
32	2	0.346687	0.346728	0.0547895	1.23878e-10
33	2	0.346687	0.346728	0.0547895	1.23878e-10
34	2	0.346666	0.346707	0.0547267	1.18482e-10
35	2	0.346666	0.346707	0.0547267	1.18482e-10
36	2	0.346628	0.346733	0.0548888	1.33341e-10
37	2	0.346666	0.346707	0.0547267	1.18482e-10
38	2	0.346726	0.346735	0.0547045	1.16137e-10
39	2	0.346666	0.346707	0.0547267	1.18482e-10
40	2	0.346628	0.346733	0.0548888	1.33341e-10

Table S4 F

Migration edge F					
**Run#**	**Starting tree#**	**W**	**W_j**	**SE_j**	**P-value**
1	1	0.48751	0.487388	0.017844	<2.22507e-308
2	1	0.487497	0.487382	0.0178219	<2.22507e-308
3	1	0.487506	0.487378	0.0178174	<2.22507e-308
4	1	0.487535	0.487406	0.0178075	<2.22507e-308
5	1	0.487506	0.487378	0.0178174	<2.22507e-308
6	1	0.487497	0.487382	0.0178221	<2.22507e-308
7	1	0.487506	0.487378	0.0178174	<2.22507e-308
8	1	0.487497	0.487382	0.0178219	<2.22507e-308
9	1	0.487511	0.487388	0.0178464	<2.22507e-308
10	1	0.487503	0.487377	0.0178124	<2.22507e-308
11	1	0.487511	0.487388	0.0178464	<2.22507e-308
12	1	0.487506	0.487378	0.0178174	<2.22507e-308
13	1	0.487503	0.487377	0.0178124	<2.22507e-308
14	1	0.487497	0.487382	0.0178219	<2.22507e-308
15	1	0.48751	0.487388	0.017845	<2.22507e-308
16	1	0.487497	0.487382	0.0178221	<2.22507e-308
17	1	0.487485	0.487377	0.0178021	<2.22507e-308
18	1	0.487511	0.487388	0.0178464	<2.22507e-308
19	1	0.487489	0.487379	0.01781	<2.22507e-308
20	1	0.487497	0.487382	0.0178221	<2.22507e-308
21	2	0.487494	0.487381	0.0178181	<2.22507e-308
22	2	0.487492	0.48738	0.0178138	<2.22507e-308
23	2	0.48751	0.487388	0.0178444	<2.22507e-308
24	2	0.487525	0.487398	0.0178291	<2.22507e-308
25	2	0.487494	0.487381	0.0178181	<2.22507e-308
26	2	0.487523	0.487398	0.0178272	<2.22507e-308
27	2	0.48751	0.487388	0.0178444	<2.22507e-308
28	2	0.487508	0.487387	0.0178421	<2.22507e-308
29	2	0.48751	0.487388	0.0178446	<2.22507e-308
30	2	0.48751	0.48738	0.017823	<2.22507e-308
31	2	0.487525	0.487398	0.0178291	<2.22507e-308
32	2	0.487507	0.487387	0.0178403	<2.22507e-308
33	2	0.487529	0.4874	0.0178369	<2.22507e-308
34	2	0.48751	0.487388	0.0178444	<2.22507e-308
35	2	0.487491	0.48738	0.0178126	<2.22507e-308
36	2	0.487494	0.487381	0.0178181	<2.22507e-308
37	2	0.487529	0.4874	0.0178369	<2.22507e-308
38	2	0.48751	0.487388	0.0178446	<2.22507e-308
39	2	0.487529	0.4874	0.0178369	<2.22507e-308
40	2	0.487493	0.487381	0.0178159	<2.22507e-308

Table S4 G

Migration edge G					
**Run#**	**Starting tree#**	**W**	**W_j**	**SE_j**	**P-value**
1	1	0.357613	0.357636	0.0231753	<2.22507e-308
2	1	0.357705	0.357662	0.0228508	<2.22507e-308
3	1	0.357705	0.35766	0.0228553	<2.22507e-308
4	1	0.357613	0.357634	0.0231757	<2.22507e-308
5	1	0.357705	0.35766	0.0228553	<2.22507e-308
6	1	0.357705	0.357662	0.0228508	<2.22507e-308
7	1	0.357705	0.35766	0.0228553	<2.22507e-308
8	1	0.357705	0.357662	0.0228508	<2.22507e-308
9	1	0.357613	0.357636	0.0231753	<2.22507e-308
10	1	0.357705	0.35766	0.0228553	<2.22507e-308
11	1	0.357613	0.357636	0.0231753	<2.22507e-308
12	1	0.357705	0.35766	0.0228553	<2.22507e-308
13	1	0.357705	0.35766	0.0228553	<2.22507e-308
14	1	0.357705	0.357662	0.0228508	<2.22507e-308
15	1	0.357613	0.357636	0.0231753	<2.22507e-308
16	1	0.357705	0.357662	0.0228508	<2.22507e-308
17	1	0.357705	0.35767	0.0228996	<2.22507e-308
18	1	0.357613	0.357636	0.0231753	<2.22507e-308
19	1	0.357705	0.35767	0.0228972	<2.22507e-308
20	1	0.357705	0.357662	0.0228508	<2.22507e-308
21	2	0.357705	0.35767	0.0228972	<2.22507e-308
22	2	0.357705	0.35767	0.0228972	<2.22507e-308
23	2	0.357613	0.357636	0.0231753	<2.22507e-308
24	2	0.357613	0.357634	0.0231757	<2.22507e-308
25	2	0.357705	0.35767	0.0228972	<2.22507e-308
26	2	0.357613	0.357634	0.0231757	<2.22507e-308
27	2	0.357613	0.357636	0.0231718	<2.22507e-308
28	2	0.357613	0.357636	0.0231753	<2.22507e-308
29	2	0.357613	0.357636	0.0231753	<2.22507e-308
30	2	0.357705	0.35766	0.0228553	<2.22507e-308
31	2	0.357613	0.357634	0.0231757	<2.22507e-308
32	2	0.357613	0.357636	0.0231753	<2.22507e-308
33	2	0.357613	0.357634	0.0231757	<2.22507e-308
34	2	0.357613	0.357636	0.0231718	<2.22507e-308
35	2	0.357705	0.35767	0.0228996	<2.22507e-308
36	2	0.357705	0.35767	0.0228972	<2.22507e-308
37	2	0.357613	0.357634	0.0231757	<2.22507e-308
38	2	0.357613	0.357636	0.0231753	<2.22507e-308
39	2	0.357613	0.357634	0.0231757	<2.22507e-308
40	2	0.357705	0.35767	0.0228972	<2.22507e-308

Table S4 H

Migration edge H					
**Run#**	**Starting tree#**	**W**	**W_j**	**SE_j**	**P-value**
1	1	0.139949	0.13997	0.0353999	3.84339e-05
2	1	0.140063	0.139974	0.03523	3.54664e-05
3	1	0.140086	0.139981	0.0352401	3.56051e-05
4	1	0.139887	0.139982	0.035153	3.41585e-05
5	1	0.140095	0.139982	0.0352558	3.58632e-05
6	1	0.140086	0.139981	0.0352401	3.56051e-05
7	1	0.140086	0.139981	0.0352401	3.56051e-05
8	1	0.140063	0.139974	0.03523	3.54664e-05
9	1	0.139949	0.13997	0.0353999	3.84339e-05
10	1	0.140095	0.139982	0.0352558	3.58632e-05
11	1	0.140063	0.139974	0.03523	3.54664e-05
12	1	0.139921	0.139978	0.0352446	3.56923e-05
13	1	0.140028	0.139979	0.0352322	3.548e-05
14	1	0.139949	0.13997	0.0353999	3.84339e-05
15	1	0.139921	0.139979	0.0352419	3.56428e-05
16	1	0.140086	0.139981	0.0352401	3.56051e-05
17	1	0.140086	0.139981	0.0352401	3.56051e-05
18	1	0.139949	0.13997	0.0353999	3.84339e-05
19	1	0.140095	0.139982	0.0352558	3.58632e-05
20	1	0.139921	0.139979	0.0352419	3.56428e-05
21	2	0.139921	0.139979	0.0352419	3.56428e-05
22	2	0.139921	0.139979	0.0352419	3.56428e-05
23	2	0.140063	0.139974	0.03523	3.54664e-05
24	2	0.140095	0.139982	0.0352558	3.58632e-05
25	2	0.140086	0.139981	0.0352401	3.56051e-05
26	2	0.140086	0.139981	0.0352401	3.56051e-05
27	2	0.140095	0.139982	0.0352558	3.58632e-05
28	2	0.140063	0.139974	0.03523	3.54664e-05
29	2	0.140095	0.139982	0.0352558	3.58632e-05
30	2	0.139949	0.13997	0.0353999	3.84339e-05
31	2	0.140095	0.139982	0.0352558	3.58632e-05
32	2	0.139949	0.13997	0.0353999	3.84339e-05
33	2	0.139949	0.13997	0.0353999	3.84339e-05
34	2	0.140095	0.139982	0.0352558	3.58632e-05
35	2	0.140063	0.139974	0.03523	3.54664e-05
36	2	0.139921	0.139979	0.0352419	3.56428e-05
37	2	0.140063	0.139974	0.03523	3.54664e-05
38	2	0.139891	0.139964	0.0351834	3.47304e-05
39	2	0.140063	0.139974	0.03523	3.54664e-05
40	2	0.139921	0.139979	0.0352419	3.56428e-05

Supplementary Figure

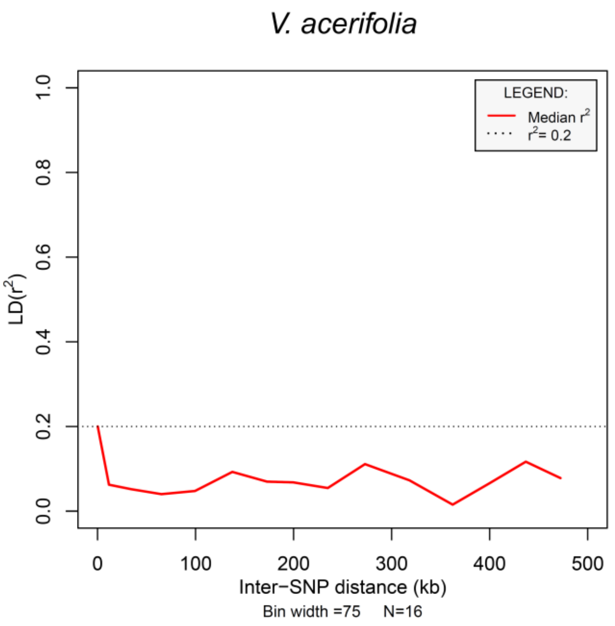
V. acerifolia
LEGEND:
Median r2
r2= 0.2
LD(r2)
0.0 0.2 0.4 0.6 0.8 1.0
0 100 200 300 400 500
Inter−SNP distance (kb)
Bin width =75 N=16

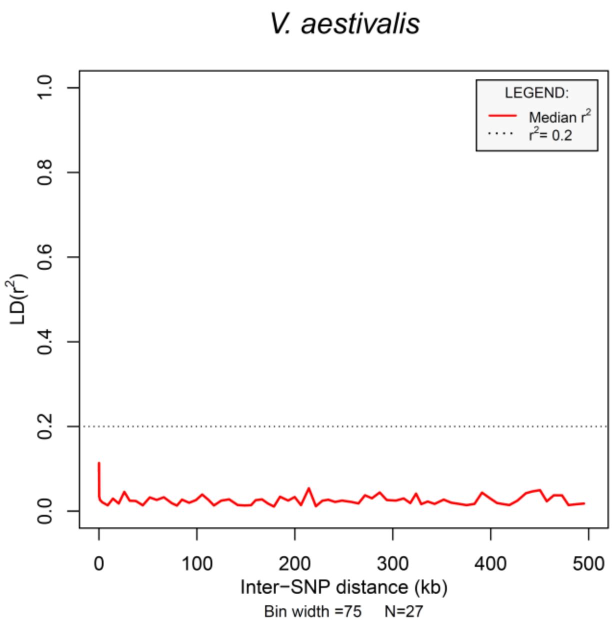
V. aestivalis
LEGEND:
Median r2
r2= 0.2
LD(r2)
0.0 0.2 0.4 0.6 0.8 1.0
0 100 200 300 400 500
Inter−SNP distance (kb)
Bin width =75 N=27

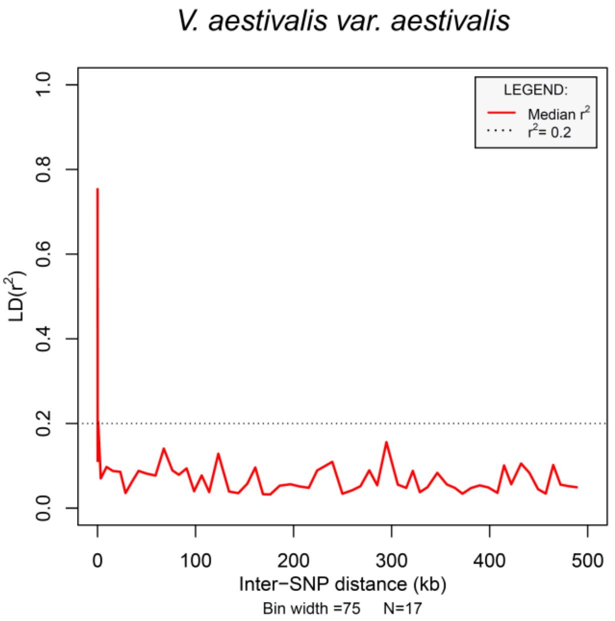
V. aestivalis var. aestivalis
LEGEND:
Median r2
r2= 0.2
LD(r2)
0.0 0.2 0.4 0.6 0.8 1.0
0 100 200 300 400 500
Inter−SNP distance (kb)
Bin width =75 N=17

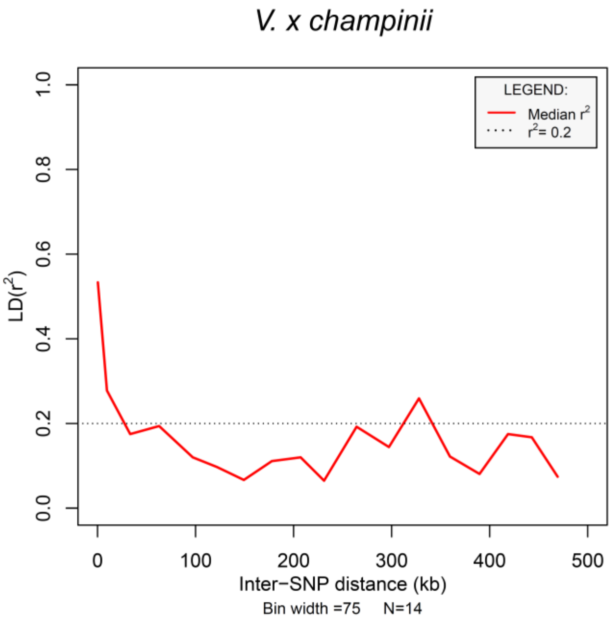
V. x champinii
LEGEND:
Median r2
r2= 0.2
LD(r2)
0.0 0.2 0.4 0.6 0.8 1.0
0 100 200 300 400 500
Inter−SNP distance (kb)
Bin width =75 N=14

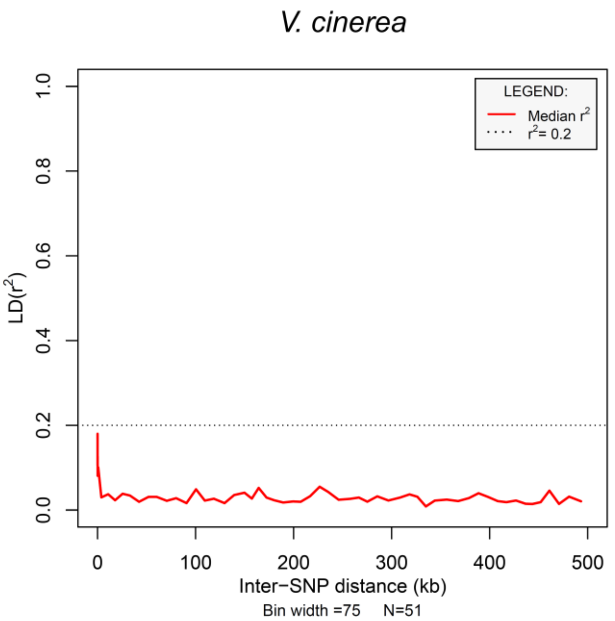
V. cinerea
LEGEND:
Median r2
r2= 0.2
LD(r2)
0.0 0.2 0.4 0.6 0.8 1.0
0 100 200 300 400 500
Inter−SNP distance (kb)
Bin width =75 N=51

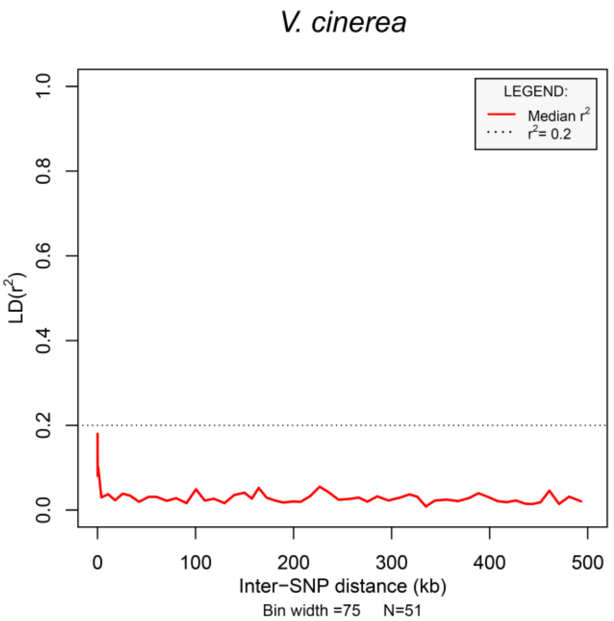
V. cinerea
LEGEND:
Median r2
r2= 0.2
LD(r2)
0.0 0.2 0.4 0.6 0.8 1.0
0 100 200 300 400 500
Inter−SNP distance (kb)
Bin width =75 N=51

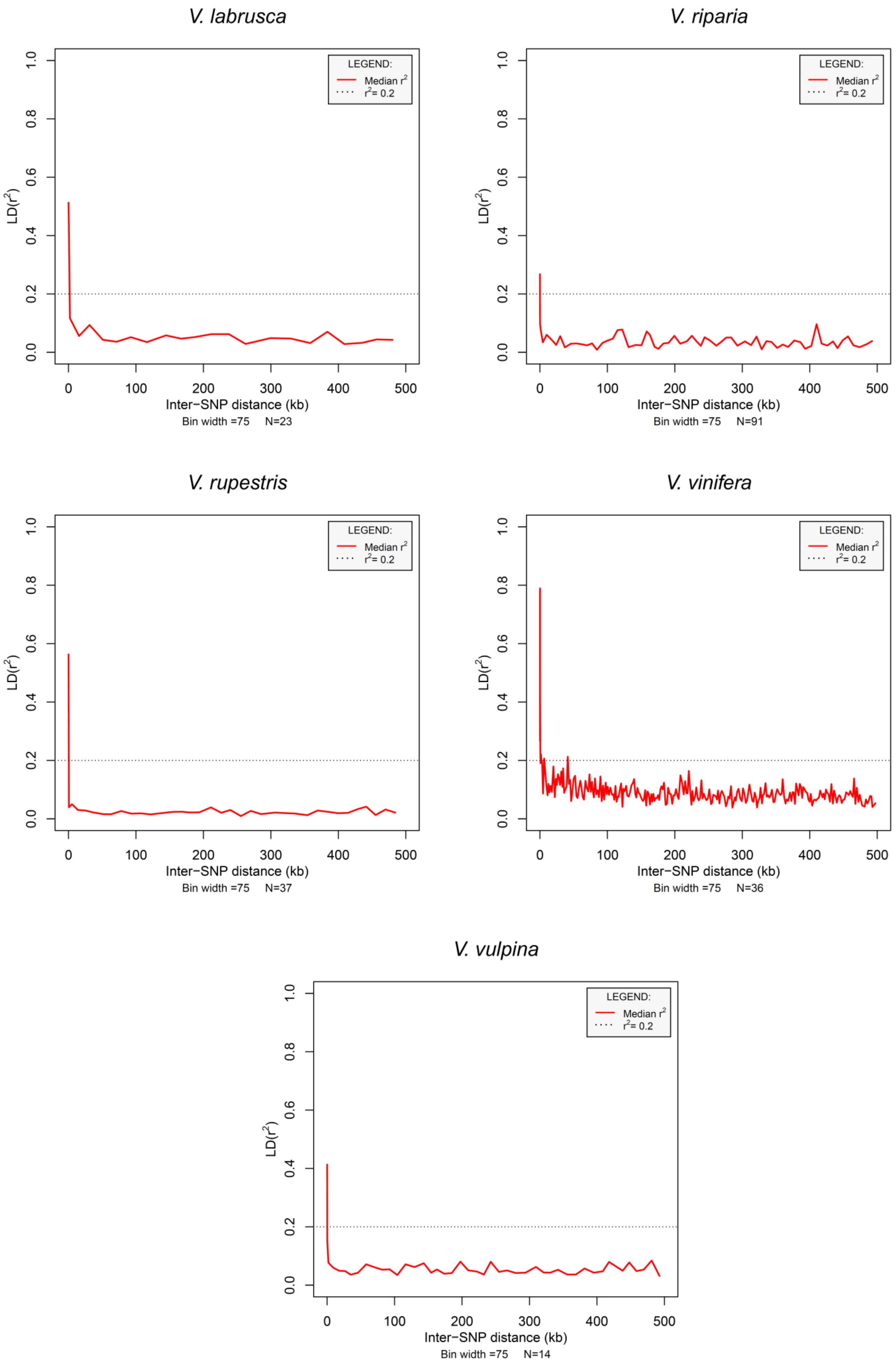
V. labrusca
V. riparia
V. rupestris
V. vinifera
V. vulpina
LEGEND:
Median r2
r2= 0.2
LD(r2)
0.0
0.2
0.4
0.6
0.8
1.0
0
100
200
300
400
500
Inter−SNP distance (kb)
Bin width =75 N=23
Bin width =75 N=91
Bin width =75 N=37
Bin width =75 N=36
Bin width =75 N=14

Figure S1: The decay of linkage disequilibrium (LD) in: *V. acerifolia*, *V. aestivalis*, *V. aestivalis var. aestivalis*, *V. champinii*, *V. cinerea*, *V. cinerea* var. *helleri*, *V. labrusca*, *V. riparia*, *V. rupestris*, *V. vinifera* subsp. *vinifera*, *V. vulpina*. LD was estimated calculating the pairwise squared correlation based on genotypic allele counts (--r^2 option in PLINK; Purcell et al. 2007) for all intrachromosomal SNP loci up to 500 kb. Each point represents the median r^2 value and the mean physical distance from bins of 75 pairwise SNP comparisons. LD decay is rapid in all species considered. N: the number of individuals sampled to estimate LD; Bin width: the number of pairwise r^2 values included in each bin; dotted black lines indicate r^2= 0.2. Physical inter-marker distance is expressed as kilobase pairs (kb).

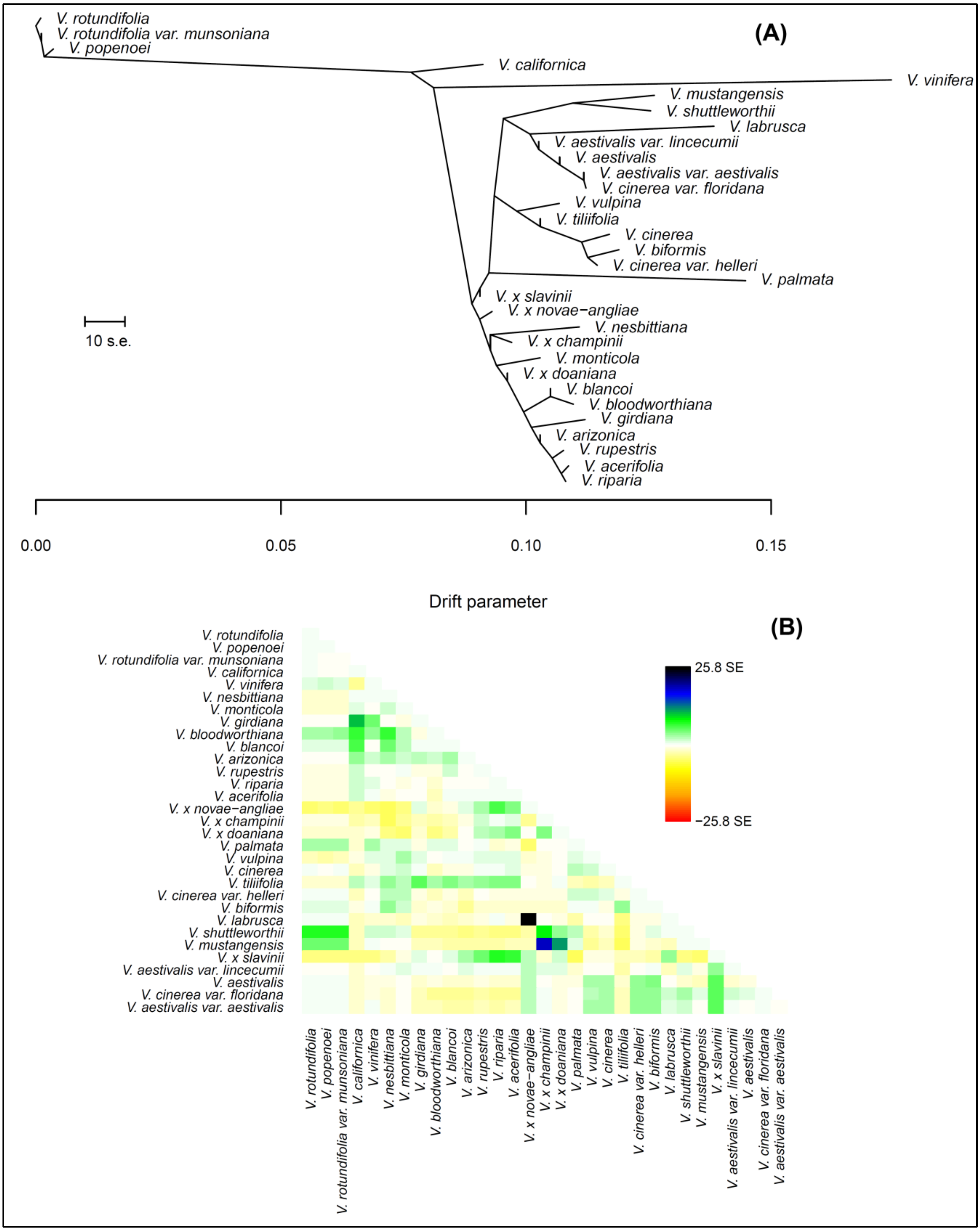

Figure S2. (**A**) One of the two ML-trees inferred by TreeMix under the strictly bifurcating model. The scale bar shows ten times the average standard error (s.e.) of the entries in the sample covariance matrix. Drift parameter is shown on the x-axis. (**B**) Scaled residuals from the fit of the model to the data. Without migration events 91.7% of the variance in relatedness between taxa was explained by the tree. Colours are described in the palette on the right.

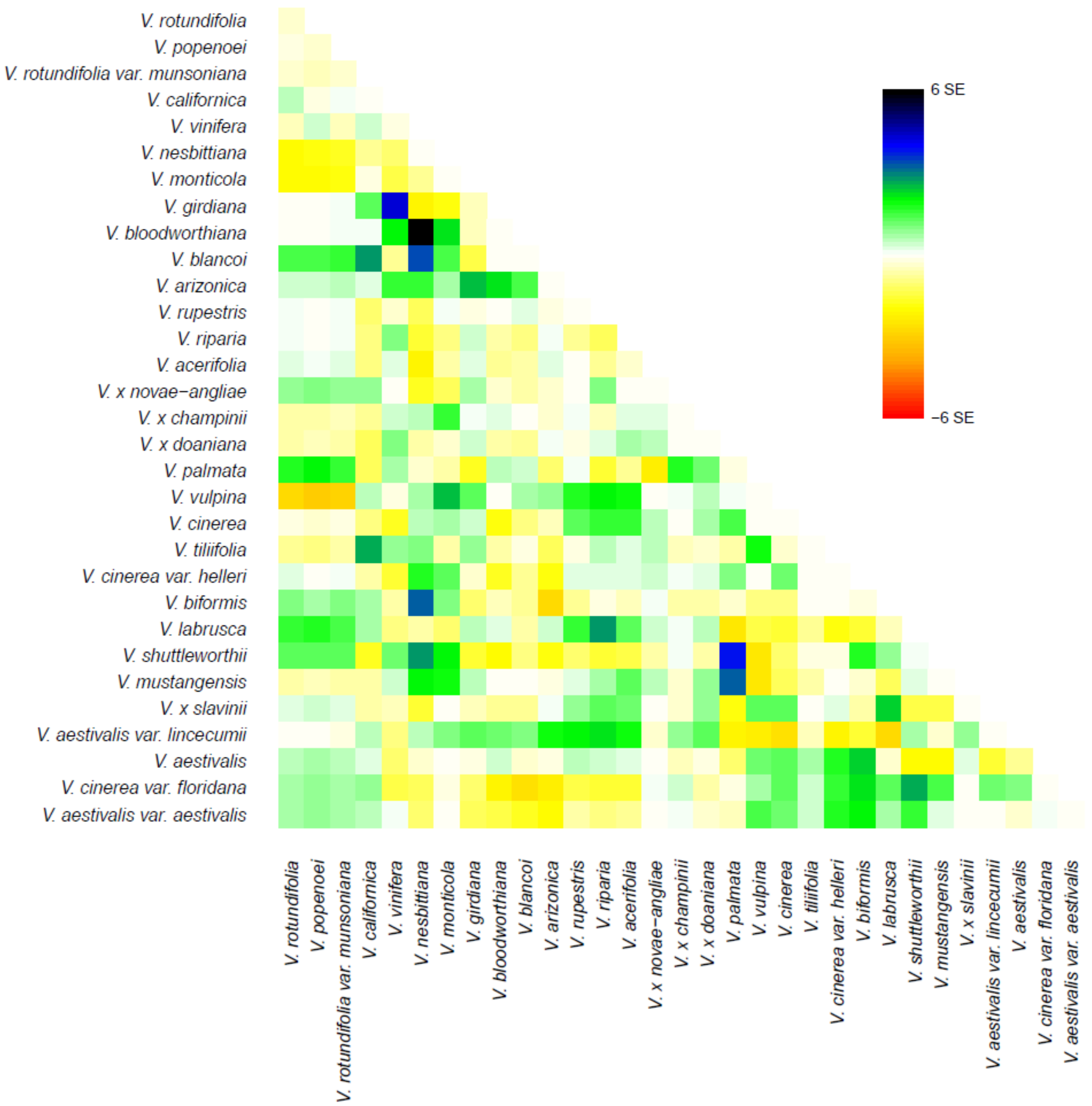

Figure S3. Scaled residuals from the fit of the model to the data. Allowing eight migration events 98.6% of the variance in relatedness between taxa was explained by the tree. Colours are described in the palette on the right.

References

Cousins, P. (1999). Genetics of Resistance to Meloidogyne incognita in Crosses of Grape Rootstocks. Dissertation, University of California, Davis.

Maul et al., (2017). Vitis International Variety Catalogue - www.vivc.de – (accessed February 2017).

Myles, S., Boyko, A. R., Owens, C. L., et al. (2011). Genetic structure and domestication history of the grape, *Proc. Natl Acad. Sci. USA*, 108, 3530–5.

Purcell, S., Neale, B., Todd-Brown, K., Thomas, L., Ferreira, M. A. R., Bender, D. et al., (2007). PLINK: a toolset for whole-genome association and population-based linkage analysis. *Am. J. Human Genetics* 81(3): 559–75.

Sawler, J., Reisch, B., Aradhya, M. K., Prins, B., Zhong, G. Y., et al. (2013). Genomics Assisted Ancestry Deconvolution in Grape. *PLoS ONE* 8(11): e80791. doi: 10.1371/journal.pone.0080791.

USDA, Agricultural Research Service, National Plant Germplasm System. 2019. Germplasm Resources Information Network (GRIN-Taxonomy). National Germplasm Resources Laboratory, Beltsville, Maryland. URL: https://npgsweb.ars-grin.gov/gringlobal/taxonomydetail.aspx?311850.
